# Supplementary material for: Predicting fracture risk in patients with chronic obstructive pulmonary disease: a UK-based population-based cohort study
Source: BMJ Open. 2019 Apr 3;9(4):e024951. doi: 10.1136/bmjopen-2018-024951 (PMC6500346; doi:10.1136/bmjopen-2018-024951)
Supplement: Supplementary data [file bmjopen-2018-024951supp001.pdf]

Appendix 1

Read code definitions for selected input variables

Variable	Read codes
COPD	H3...00, H3...11, H31..00, H310.00, H310000, H310z00, H311.00, H311000, H311100, H311z00, H312.00, H312000, H312011, H312100, H312300, H312z00, H313.00, H31y.00, H31y100, H31yz00, H31z.00, H32..00, H320.00, H320000, H320100, H320200, H320300, H320311, H320z00, H321.00, H322.00, H32y.00, H32y000, H32y100, H32y111, H32y200, H32yz00, H32z.00, H36..00, H37..00, H38..00, H39..00, H3A..00, H3y..00, H3y..11, H3z..00, H3z..11
Osteoporosis	585O.00, 58E4.00, 58E8.00, 58EA.00, 58EE.00, 58EG.00, 58EK.00, 58EM.00, 58ES.00, 58EV.00, 7230A, 7230B, 7230D, 7230PM, 7230PT, N330.00, N330000, N330100, N330200, N330300, N330400, N330500, N330600, N330700, N330800, N330900, N330A00, N330B00, N330C00, N330D00, N330z00, N331200, N331300, N331400, N331500, N331600, N331800, N331900, N331A00, N331B00, N331M00, N331N00, NyuB000, NyuB100, NyuB200, NyuB800
Antiresorptive treatment *(drug code)*	97138998, 99158998, 99158997, 97139998, 96920998, 96789998, 93478998, 97140998, 97218998, 93975992, 83457998, 97064992, 83456998, 96897998, 96020992, 96901998, 95879992, 98249990, 97031992, 98581990, 99018990, 98198990, 62945979, 96737998, 97066992, 97051992, 97780990, 98199990, 61594979, 99261990, 96604992, 92004979, 97248990, 99263990, 94089992, 93127992, 94756992, 91526998, 89828998, 88144998, 88144997, 88225998, 89434998, 93502998, 99862998, 95304998, 93228997, 96904998, 93228998, 95304996, 99862997, 95304997, 93228996, 99864998, 91997998, 91998998, 87933998, 81073998, 61612979, 87155998, 87154998, 88542998, 91378998, 82066998,

Antiresorptive treatment *(drug code)*	82065998, 81256998, 81255998, 91190996, 89518998, 91190998, 91191998, 86599998, 91190997, 91191997, 93692990, 81472998, 94276990, 93827990, 92431990, 94161990, 93610990, 94245990, 61524979, 99883979, 93828990, 99867979, 95572998, 99758998, 96764998, 97398992, 95244990, 89367998, 86562998, 86561998, 87645998, 87644998, 86079998, 86076998, 91533998, 87151998, 81270998, 91027998, 93617996, 93618996, 93618997, 93617997, 90527998, 86566998, 91028998, 87137998, 87136998, 91674998, 86564998, 86567998, 87135998, 93089979, 99357998, 84212998, 84691998, 89021998, 91764998, 90551998, 91763998, 81869998, 91764997, 91763997, 89354979, 92813997, 93402998, 92813998, 98527996, 93403996, 98527998, 93403998, 93402996, 84531998, 58602979, 87606998, 85936998, 81112998, 97865998, 85935998, 81111998, 76983978, 83078978

Appendix 2

METHODS

Potential confounders

For smoking status, alcohol use, MRC Dyspnoea scale, and a list of prescription drugs, the most recent record within 1 year (before and after) of index date were used. A BMI record within 2 years (before and after) of index date was used.

Where possible BMI was calculated from height and weight records, for patients with a missing BMI record. The BMI was subsequently categorised (underweight: <18.5 kg/m^2, normal: 18.5-<25 kg/m^2, overweight: 25-<30 kg/m^2, obese: >30 kg/m^2).

Having received at least one prescription for inhaled corticosteroids, anti-epileptics, antidepressants, oestrogen-only Hormone Replacement Therapy (HRT) and osteoporosis medications, within 1 year (before and after) of index date were considered as risk factors.

Prediction tools – Input variables

The respective variable definitions as outlined in the algorithms for the prediction tools were used.

Smoking status – In QFracture®, three current smoking categories are provided according to the number of cigarettes smoked daily[1]. To avoid the bias of categorising patients in one of the outlying categories, "current smokers" with no documented number of cigarettes smoked were assigned to the middle category "10-19 cigarettes daily" as done in a recent publication [2]. For FRAX®'s two-category smoking status, former smokers were assigned to the "non-smoker" category as was done in the cohorts used to develop FRAX®.[3]

Alcohol consumption – similarly, for alcohol use in QFracture®, alcohol drinkers with no documented unit/day intake were assigned to "moderate (3-6units/day)".

Missing values for BMI, smoking status, and alcohol use were imputed by multiple imputation using all predictors, resulting in twenty imputed datasets.[4] A complete case sensitivity analysis without imputed variables was also performed (Appendix 3).

References

1 ClinRisk Ltd. QFracture-2016® risk calculator. http://www.qfracture.org/ (accessed 20 Sep 2017).

2 Dagan N, Cohen-Stavi C, Leventer-Roberts M, *et al.* External validation and comparison of three prediction tools for risk of osteoporotic fractures using data from population based electronic health records: retrospective cohort study. *BMJ* 2017;**356**:i6755. doi:10.1136/BMJ.I6755

3 Kanis JA, Oden A, Johnell O, *et al.* The use of clinical risk factors enhances the performance of BMD in the prediction of hip and osteoporotic fractures in men and women. *Osteoporos Int* 2007;**18**:1033–46. doi:10.1007/s00198-007-0343-y

4 Horton NJ, Lipsitz SR. Multiple Imputation in Practice. *Am Stat* 2001;**55**:244–54. doi:10.1198/000313001317098266

Appendix 3

Fracture risk prediction tools in COPD (Complete case analysis)

Of the 72,559 patients aged 40-90 years with COPD and no prior diagnosis of osteoporosis or prescription of any anti-resorptive treatment, 41,879 (57.7%) of patients had complete data. Amongst the patients with complete data, 2,649 (6.3%) experienced a MOF and 806 (1.9%) experienced hip fracture.

Both risk tools had about the same discriminatory accuracy as that obtained from the entire cohort with imputed data. The AUC for hip fracture was 75.6%, 95% CI 74.0% to 77.1% for FRAX® and 75.6%, 95% CI 74.0% to 77.2% for QFracture®. FRAX® maintained a higher accuracy for MOF (71.6%, 95% CI 70.6% to 72.6%) than QFracture® (61.1%, 95% CI 60.0% to 62.2%).

Appendix 4

Figure E1: Study population flow diagram

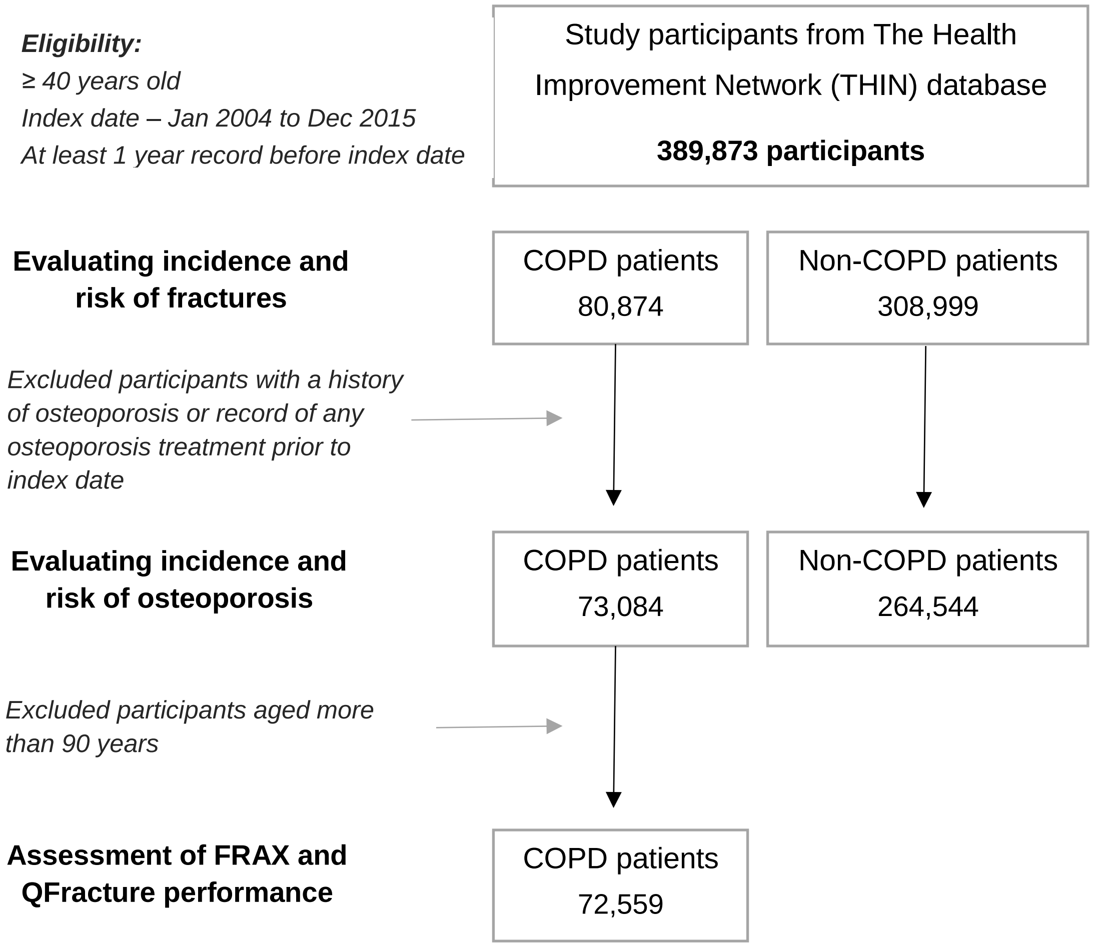

Appendix 5

Table E1: Risk of osteoporosis in patients with COPD compared with non-COPD patients

Descriptor	HR (95% CI)	Fully adjusted HR (95% CI)
**COPD**		
Non-COPD subjects	Reference	Reference
COPD patients	1.96 (1.87 – 2.06)	1.13 (1.05 – 1.22)
**Charlson Comorbidity Index**		
Score 0	Reference	Reference
Score 1	1.27 (1.18 – 1.36)	1.14 (1.06 – 1.23)
Score 2	1.34 (1.24 – 1.44)	1.27 (1.17 – 1.37)
Score 3	1.41 (1.28 – 1.55)	1.29 (1.17 – 1.42)
Score 4 & more	1.48 (1.33 – 1.64)	1.44 (1.29 – 1.61)
**Body Mass Index** (kg/m^2)		
Underweight (<18.5)	1.93 (1.64 – 2.27)	1.91 (1.63 – 2.25)
Normal (18.5 – 24.9)	Reference	Reference
Overweight (25 – 29.9)	0.64 (0.60 – 0.69)	0.63 (0.58 – 0.67)
Obese (≥ 30)	0.47 (0.43 – 0.51)	0.45 (0.41 – 0.48)
No record	0.50 (0.46 – 0.53)	0.57 (0.52 – 0.61)
**Smoking status**		
Never	Reference	Reference
Ex	1.01 (0.95 – 1.08)	1.02 (0.95 – 1.09)
Current	1.23 (1.13 – 1.33)	1.15 (1.06 – 1.25)
Unknown	0.69 (0.64 – 0.74)	0.77 (0.71 – 0.83)
**Oral Corticosteroid Use**		
Unexposed	Reference	Reference
Exposed	2.79 (2.56 – 3.05)	1.91 (1.73 – 2.10)
**Inhaled Corticosteroid Use**		
No	Reference	Reference
Yes	1.35 (1.26 – 1.45)	1.24 (1.15 – 1.34)

HR – Hazard ratio; CI – Confidence interval

HR – Cox regression model derived HR adjusted for age, sex, and GP practice

The fully adjusted Hazard Ratio (aHR) was 1.13, 95% CI 1.05 to 1.22, $p<0.0001$ – the multivariable Cox regression model derived aHR was adjusted for age, sex, GP practice, Charlson comorbidity index, body mass index, smoking status, inhaled corticosteroid use, and cumulative oral corticosteroid use.

Appendix 6

Table E2: Baseline characteristics of patients with COPD aged 40-90 years with no prior diagnosis of osteoporosis or prescription of any anti-resorptive treatment

**Descriptor**	**COPD patients**	
	n **=** 72,559	**%**
**Mean age at index date (years, SD)**	66.1 (10.7)	
**Sex**		
Female	31,885	43.9
**MRC Dyspnoea Scale** (1 Year either side of diagnosis)		
1	8,882	12.2
2	17,718	24.4
3	9,257	12.8
4 & 5	4,346	6.0
No record	32,356	44.6
**Charlson Comorbidity Index Score**		
0	0	0
1	38,573	53.2
2	11,953	16.5
3	11,110	15.3
≥ 4	10,923	15.1
**Body Mass Index (BMI) (kg/m^2)**		
Underweight (< 18.5)	2,730	3.8
Normal (18.5 – 24.9)	21,791	30.0
Overweight (25 – 29.9)	21,504	29.6
Obese (≥30)	17,627	24.3
No BMI	8,907	12.3
**Smoking status** (1 Year either side of diagnosis)		
Never smoked	7,062	9.7
Ex-smoker	33,810	46.6
Current smoker	29,949	41.3
Unknown	1,738	2.4
