# Supplementary material for: Predicting fracture risk in patients with chronic obstructive pulmonary disease: a UK-based population-based cohort study
Source: BMJ Open. 2019 Apr 3;9(4):e024951. doi: 10.1136/bmjopen-2018-024951 (PMC6500346; doi:10.1136/bmjopen-2018-024951)
Supplement: Supplementary file 2 [file bmjopen-2018-024951.draft_revisions.pdf]

BMJ Open is committed to open peer review. As part of this commitment we make the peer review history of every article we publish publicly available.

When an article is published we post the peer reviewers' comments and the authors' responses online. We also post the versions of the paper that were used during peer review. These are the versions that the peer review comments apply to.

The versions of the paper that follow are the versions that were submitted during the peer review process. They are not the versions of record or the final published versions. They should not be cited or distributed as the published version of this manuscript.

BMJ Open is an open access journal and the full, final, typeset and author-corrected version of record of the manuscript is available on our site with no access controls, subscription charges or pay-per-view fees (http://bmjopen.bmj.com).

If you have any questions on BMJ Open's open peer review process please email editorial.bmjopen@bmj.com

BMJ Open

Predicting Fracture Risk in Patients with COPD using The Health Improvement Network (THIN)

Journal:	*BMJ Open*
Manuscript ID	bmjopen-2018-024951
Article Type:	Research
Date Submitted by the Author:	27-Jun-2018

Complete List of Authors:	Akyea, Ralph; University of Nottingham, Nottingham Respiratory Research Unit, NIHR Nottingham Biomedical Research Centre; University of Nottingham, Division of Epidemiology & Public Health<br>McKeever, Tricia; University of Nottingham, Nottingham Respiratory Research Unit, NIHR Nottingham Biomedical Research Centre; University of Nottingham, Division of Epidemiology & Public Health<br>Gibson, Jack; University of Nottingham, Division of Epidemiology & Public Health<br>Scullion, Jane; Institute for Lung Health, University Hospitals of Leicester Glenfield Site<br>Bolton, Charlotte; University of Nottingham, Nottingham Respiratory Research Unit, NIHR Nottingham Biomedical Research Centre

Keywords:	Fracture, COPD, fracture risk prediction tool, osteoporosis

SCHOLARONE™
Manuscripts

For peer review only

Predicting Fracture Risk in Patients with COPD using The Health Improvement Network (THIN)

Ralph K Akyea[1, 2]; Tricia M McKeever[1, 2]; Jack E Gibson[2]; Jane E Scullion[3]; Charlotte E Bolton[1]

Authors affiliations:

[1] Nottingham Respiratory Research Unit, NIHR Nottingham Biomedical Research Centre, School of Medicine, University of Nottingham, UK.

[2] Division of Epidemiology and Public Health, School of Medicine, University of Nottingham, UK.

[3] University Hospitals of Leicester Glenfield Site, Institute for Lung Health, Leicester, UK.

Corresponding author information:

Dr Charlotte E Bolton,

Nottingham Respiratory Research Unit, NIHR Nottingham Biomedical Research Centre, School of Medicine, University of Nottingham, City Hospital Campus, Hucknall Road, Nottingham, NG5 1PB, UK.

Tel: +44 (0)115 8231710

Email: charlotte.bolton@nottingham.ac.uk

Keywords:

Fracture, osteoporosis, COPD, fracture risk prediction tool

Word counts:

Abstract: 259

Main text: 2,795

For peer review only

ABSTRACT

Objectives To assess incidence of hip fracture or major osteoporotic fractures (MOF) in patients with COPD compared to non-COPD subjects and to evaluate the use and performance of fracture risk prediction tools in patients. To assess recorded osteoporosis diagnosis.

Design A population-based cohort study

Setting UK General Practice health records from The Health Improvement Network database

Participants Patients with an incident COPD diagnosis from 2004-2015 and age, sex and general practice matched non-COPD subjects were studied.

Outcomes Incidence of fracture; accuracy of fracture risk prediction tools in COPD; prevalence and incidence of osteoporosis.

Methods: Stratified Cox proportional hazards models (stratified matched cohort analyses) were used. The discriminatory accuracy (area under the receiver operating curve [ROC]) of fracture risk prediction tools in COPD was assessed.

Results There was an increased risk of fracture in patients with COPD but this was largely mediated through oral corticosteroid use, BMI and smoking. Retrospectively calculated discriminatory accuracies for major osteoporotic fracture were FRAX®: 71.4% (95% CI: 70.6 to 72.2%), QFracture®: 61.4% (95% CI: 60.5 to 62.3%) and for hip fracture both 76.1% (95% CI: 74.9 to 77.2%). Prevalence of osteoporosis was greater for patients (5.7%) compared to non-COPD subjects (3.9%), $p<0.001$. In those without former osteoporosis, patients (n=73,084) had an increased osteoporosis incidence compared to non-COPD subjects (n=264,544), (adjusted hazard ratio, 1.13, 95% CI 1.05 to 1.22).

Conclusion COPD patients are at increased risk of fractures and osteoporosis. Despite this, there is no systematic assessment of fracture risk in clinical practice. Fracture risk tools identify those at high-risk of fracture in patients with COPD.

For peer review only - http://bmjopen.bmj.com/site/about/guidelines.xhtml

Strengths and limitations of this study

- This research was population-based using electronic health records representative of the UK population with a substantial duration of follow-up.
- A wide range of potential confounders were also evaluated and adjusted for in the analyses.
- For the assessment of the fracture prediction tools, the population of patients with COPD used was large, with many fracture events, included both men and women and is representative of the UK population.
- Whilst coded osteoporosis diagnosis appears under-reported in COPD compared to where osteoporosis is systematically sought in patients with COPD, this was a secondary outcome. Further the under-reporting is worthy of mention.

For peer review only

INTRODUCTION

Osteoporosis in both male and female patients with COPD is firmly established as one of the core comorbid conditions.[1,2] Over the last decade, it has become clear that osteoporosis is not just an end-stage COPD problem[3] nor just in those on maintenance oral corticosteroids, but it also occurs in a large proportion of those with mild-moderate airflow obstruction and even in steroid naïve patients.[4,5] The Global Initiative for Chronic Obstructive Pulmonary Disease (GOLD strategy recommends that osteoporosis co-existence should be considered in COPD [1] and the UK National Institute for Health and Care Excellence (NICE) Guidelines on osteoporosis considers COPD as a secondary cause of osteoporosis encouraging use of fracture prediction tools.[6]

The causes for osteoporosis in COPD are likely multiple and cumulative, including age, smoking exposure, inactivity, low body mass index (BMI), systemic inflammation and the frequent use of oral corticosteroids.[7] The clinical implications of osteoporosis include increased risk of fractures, poor quality of life, pain and further deterioration in lung function.[8,9] Osteoporosis can also remain undiagnosed as asymptomatic for many years.[10] Fractures are a function of trauma sustained, such as falls which are common in COPD [11], and the quality and architecture of bone. Fractures contribute further pain, poor quality of life, increased mortality and confer a substantial economic burden on health systems, patients and their families.[12,13] Given this, the individual risk of a future fracture in patients with COPD is crucial to determine in patient care and to treat accordingly.

Fracture risk prediction tools based on clinical and personal characteristics have been developed over the years to guide investigation and management of those identified to be at high risk of osteoporotic fractures, worldwide. These include for the UK (and many other regions), FRAX® and QFracture®.[6]

The full extent of fracture risk assessment in patients with COPD is not fully established. The aim of this study was to evaluate fracture in patients with COPD compared to non-COPD subjects together with the use of and the performance of fracture risk prediction tools in patients with COPD. Further, to assess the coding of osteoporosis in patients with COPD and non COPD subjects.

For peer review only

METHODS

Information for this cohort study was obtained from The Health Improvement Network (THIN), an anonymised primary care database representing 6.2% of the total UK population.[14]

The study population consisted of patients 40 years and over with a new Read coded COPD diagnosis during the data collection period 1/1/04-31/12/15, with at least 1 year of record prior to COPD diagnosis.[15] Each patient was matched by age, gender and GP practice to up to four subjects without a history of COPD to generate a matched cohort and assigned the same index date.

Follow up was from the index date to the first record of either the occurrence of the outcome of interest (fracture/osteoporosis), the date of transfer of the patient out of the practice area, death or end of THIN data collection. Read coded diagnoses for osteoporosis and read coded hip fracture or major osteoporotic fractures (MOF) (fracture of the hip, proximal humerus, forearm or clinically symptomatic vertebra/spine) were ascertained.

A series of explanatory variables [6,16] determined at baseline (prior to or at index date) included: Charlson Comorbidity Index (CCI) score,[17] Townsend social deprivation score, fall, prior fractures, parental history of fall/osteoporosis, relevant comorbidities and secondary causes of osteoporosis as defined in the FRAX® questionnaire.[18] Records for smoking status, alcohol use, MRC Dyspnoea scale, BMI, and use of specific prescription drugs were restricted to a defined time period. Oral corticosteroid (OCS) use was considered as a time-dependent variable with exposed and non-exposed periods. Exposed periods started from prescription date until the first gap of more than 90 days between prescriptions. OCS prescriptions issued within 90 days prior to index date were considered as part of exposed periods.

Input variables included clinical status, prescription drug use, and demographic characteristics, according to the variables/definitions used in both FRAX® and QFracture® tools,[18,19], additional detail on the method is provided in an online data supplement *(Appendix 1)*. Imputation was used for missing variables.

For peer review only

The 10-year risk score for hip fracture and MOF according to QFracture® (version 2017.0.0.0) and FRAX® for UK without BMD information (desktop version 3.12) were calculated for patients with COPD, aged 40-90 years old.

Statistical analyses

Incidence rates were calculated for both groups using time-dependent Cox proportional hazards regression to estimate hazard ratios (HRs) of osteoporosis and fracture risks, with OCS treated as a time-dependent variable. Confounders were included in the final model when independently changing the HRs for osteoporosis/fracture by at least 5%. A former osteoporosis diagnosis or antiresorptive treatment prior to COPD diagnosis excluded that subject from analyses related to either osteoporosis incidence or risk. In addition to evaluating incidence in the whole cohort, separate sub-analyses excluded a) patients with COPD and no documented smoking history together with their matched non-COPD subjects and b) those with no prior record of osteoporosis.

To evaluate FRAX® and QFracture®, the outcome was treated as a binary variable (fracture or no fracture). Fracture risk probabilities were categorised based on recommended treatment thresholds (≥ 20% for MOF and ≥3% for hip fracture).[20] To evaluate the overall ability of each tool to discriminate (performance) between those at low and high risks, the area under the receiver operating characteristic (ROC) curve was calculated. Sensitivity, specificity, positive and negative predictive values were calculated. Survival analysis was performed and Kaplan-Meier plots comparing the fracture incidence were generated.

All statistical analyses were performed using Stata 15.0 (StataCorp LP).

Patient involvement

The results and implications of previous research from the team on systematic assessment of osteoporosis in patients with COPD [4] has been discussed extensively in previous patient meetings. Whilst this and other literature has strengthened the GOLD strategy recommendations,[1] evaluation of clinical services would suggest systematic assessment is not done in patients. More recently, patients with COPD out-patient clinics have approached the principal investigator at the time of their "ad hoc osteoporosis" diagnosis to ask why this was not investigated at or closer to COPD diagnosis and how osteoporosis could be assessed. This has led to the development of this grant application with significant patient input in the design and

For peer review only

context. The results have been discussed back with representatives on the respiratory research panel. Given the implications for clinical practice, the findings have been discussed extensively at the PPI meeting and a Breathe Easy meeting in early 2018. A lay summary has been developed for the patient newsletter (n>700) and website. In the meantime, members of the respiratory research panel are assisting the PI in planning future work regarding implementation.

For peer review only

RESULTS

The baseline characteristics are shown in Table 1. A total of 80,874 eligible patients with COPD and 308,999 matched non-COPD subjects were identified. The median follow-up period was 5 years for both patients and non-COPD subjects.

Osteoporosis at index date and incidence

Within 1 year (before and after) of the index date, 1,504 (1.86%) patients with COPD had a new recorded diagnosis of osteoporosis compared to 3,059 (1.12%) in matched non-COPD subjects, p <0.001. 3,186 (3.94%) of patients with COPD had a diagnosis of osteoporosis more than a year prior to index date compared to 8,822 (2.86%) for the matched controls p <0.001.

1,457 (1.80%) patients with COPD compared to 3,694 (1.20%) non-COPD subjects, had a record of any diagnostic assessment for osteoporosis, recorded within 1 year (before and after) of the index date, (p<0.001).

Demographics remained similar after excluding those with former osteoporosis. Patients with COPD (n=73,084) compared to non-COPD subjects (n=264,544) were significantly more likely to have incident diagnosis of osteoporosis (crude hazard ratio (HR), 1.96; 95% confidence interval [CI] 1.87 to 2.05; *Appendix 2*).

Incidence of Fracture

There was a significantly increased risk of both MOF, crude hazard ratio of 1.60 (95% CI 1.52 to 1.69) and hip fractures: 1.67 (95% CI 1.56 to 1.80) in patients with COPD compared to non-COPD subjects in the unadjusted model, which remained significant after adjustment for age, gender and GP practice. In the fully adjusted models the association were diminished (Table 2). Smoking status altered the effect between COPD and fracture the most, followed by BMI, CCI score and oral corticosteroid.

Sensitivity analysis with participants with no former osteoporosis showed similar results. The risk of major osteoporosis fracture was also similar when evaluated in only patients with COPD with a prior history of smoking and their matched controls. However, here, the risk of hip fracture remained significantly increased in the

For peer review only

adjusted model compared to non-COPD subjects (aHR, 1.13; 95% CI 1.004 to 1.280; p-value: 0.043).

Fracture risk prediction tools in COPD

Only 1074 (1.33%) of patients with COPD had a FRAX® assessment READ coded ever documented in the records and 12 patients had a READ coded QFracture® assessment. Within 1 year (before and after) of index date, 248 (0.31%) of patients with COPD had a FRAX® and only 1 patient a QFracture®.

The final population for the discriminatory accuracy analysis comprised 72,559 patients aged 40-90 years with COPD and no prior diagnosis of osteoporosis or prescription of any anti-resorptive treatment (*demographics in Appendix 3*). This included 4,605 (6.4%) who experienced a MOF and 1,444 (2.0%) who experienced hip fracture.

When the FRAX® and QFracture® scores were calculated for patients with COPD, for hip fracture 29,035 (40.0%) had a risk ≥3% using FRAX® and 33,065 (45.6%) using the QFracture®. For MOF, 6,221 (8.6%) of patients had a risk ≥20% using FRAX® and 9,546 (13.2%) using QFracture®.

Both risk tools had a similar discriminatory accuracy for hip fracture (FRAX® 76.1%, 95% CI 74.9 to 77.2% and QFracture® 76.1%, 95% CI 74.9 to 77.2%). FRAX®, however, had a higher accuracy for MOF (71.4% 95% CI 70.6% to 72.2%) than QFracture® (61.4% 95% CI 60.5% to 62.3%).

The discriminatory accuracies were better in women than men. The performance of the prediction tools was similar in the patients aged 50-90 years compared to 40-90-year olds. Table 3 shows the results for the sensitivity, specificity, positive and negative predictive values assessed for the performance of the prediction tools at ≥3% risk probability for hip fracture and ≥20% risk probability for major osteoporotic fractures.

The Kaplan-Meier plots for time to first MOF for QFracture® and FRAX® are presented in Figure 1.

For peer review only - http://bmjopen.bmj.com/site/about/guidelines.xhtml

DISCUSSION

Using UK primary care electronic health records, we have reported on the burden of fractures in patients with COPD with both hip and major osteoporotic fractures increased in patients with COPD compared to age, gender and GP surgery matched subjects. Despite the increased fracture risk and recommendations in the NICE osteoporosis guidelines, fracture risk prediction tools are rarely coded. However, where the risk score was retrospectively calculated, the risk prediction tools identify those at risk of hip fracture or MOF. Therefore, fracture risk prediction and subsequent targeted therapy and management could transform multi-morbidity management of COPD. In addition, we report that the prevalence and incidence of osteoporosis, a risk for fracture, in patients with COPD, is far greater than in non-COPD subjects.

Prevalence of osteoporosis varies widely in the different studies of patients with COPD. This is mainly dependent on the severity of COPD,[4,5] whether osteoporosis was systematically sought or self-reported [4,21], and whether patients included were on oral corticosteroids.[3] A prevalence of 23-32% has been reported where BMD was systematically performed [22].[4], while 14% of patients with COPD self-reported osteoporosis compared to 5% in those without COPD.[21] The prevalence of coded osteoporosis in the GP health records was, however, far lower at 5.7% than the reported prevalence from clinical studies when osteoporosis and BMD are systematically assessed. This raises the question of subclinical, undiagnosed disease leading to a missed opportunity for intervention and strengthening the need for a systematic assessment especially when cost-efficient anti-resorptive treatment is available.[23]

There is growing consensus on COPD being a secondary cause of osteoporosis, including within the NICE clinical guideline on osteoporosis where fracture risk prediction tools are recommended, yet in practice seem rarely done.[6] Whilst osteoporosis in itself leads to pain and poor quality of life,[24] ultimately osteoporosis treatment aims to reduce the risk of fracture.[23,25] Risk factors for fracture include osteoporosis but also falls, which, are greater in patients with COPD.[11,26] Whilst the increased risk of fractures in COPD has previously been considered,[27] they have not assessed incidence from time of COPD diagnosis or only reported as part of a larger study of post-menopausal women [28] or analysed the history of obstructive airway disease (both COPD and asthma together) before

For peer review only - http://bmjopen.bmj.com/site/about/guidelines.xhtml

the index date of osteoporotic fracture in both cases and controls over the age of 18 years.[29]

Little is known about the use of fracture risk assessment tools in patients with COPD. A number of validation studies have performed independent assessments to predict subsequent fracture in the general population.[32,33] The studies differ widely in sample size, methodology, and techniques used to assess performance.[34] Discrimination for FRAX® (without BMD incorporation) and QFracture® have both been reported as good.[32,35,36] The results from this COPD study are comparable to the general population validation studies.[32,35,36] The discrimination from our study was better in women and for hip fracture as it is in the general population studies – both associated with the greatest morbidity and mortality.[37] The discrimination appeared similar within the 40-90 and 50-90 year-old groups. Despite the two tools having differences in their approach to calculating fracture risks, both predict fractures satisfactorily in patients with COPD and will thus be helpful in selecting high-risk patients. Available fracture prevention therapy (anti-resorptive agents) are very effective, safely yielding 40-60% reduction in the risk of fracture.[25] These medications are cost-effective in high-risk patients –reduces morbidity, mortality and health care cost associated with osteoporotic fractures.[23] These fracture prediction tools could be integrated into COPD annual assessments or diagnosis to identify patients at high fracture risk, assist in selecting efficacious treatment and provide long-term follow-up with serial assessments. Though the optimal pathways for this integration is required.

The use of oral corticosteroids has been considered to be a major contributory factor in the development of osteoporosis. However, osteoporosis has been reported in patients with no oral corticosteroid use.[4,5] Other known osteoporosis risk factors are also likely to contribute in patients with COPD including smoking, a low BMI, physical inactivity and systemic inflammation. Some of these risk factors could be moderated through education, smoking cessation, pulmonary rehabilitation and lifestyle changes.[30,31] Recognition of the scale and impact of fracture risk draws further necessary attention to these interventions to aim to prevent and reduce risks, alongside appropriate pharmacotherapy.

The study had several strengths in its methods, analyses, findings, and implications for clinical practice. Firstly, this research was population-based and compared patients with COPD with age-sex matched control subjects from the same general practice. Its external validity and hence generalisability was high because THIN

For peer review only

database is representative of the UK population. There was a substantial duration of follow-up. A wide range of potential confounders were also evaluated and adjusted for in the analyses.

For the assessment of the fracture prediction tools, the population of patients with COPD used was large, with many fracture events, included both men and women and is representative of the UK population. This minimised the likelihood of a selection bias. The assessments of the prediction tools were done using the same population, therefore minimising the effect of confounding for a difference in performance. We are presently not aware of studies that have determined the performance of the recommended fracture prediction tools in the sub-population of patients with COPD. The dataset was using UK electronic health records but is likely representative of other countries in representing the scale of the problem and the utility of the risk prediction scores.

Regarding limitations, some variables might be subject to information or reporting bias, including patient reported alcohol intake, use of cigarettes or their awareness of relevant family history. The possibility of residual confounding can also not be excluded as risk factors such as physical activity, diet and ethnicity could not be adjusted for in the analyses. An accepted definition of fractures types was used; however, it is difficult to determine the cause of fracture based simply on fracture site, with no additional information. Unlike studies which assess BMD systematically, this is not currently done in clinical practice, nor are the fracture risk scores routinely calculated as highlighted here. Therefore, the incidence of osteoporosis based on clinical codes likely reflects an underestimation of the true increased incidence/risk of osteoporosis.

In summary, despite validated fracture risk prediction tools, there was very little assessment of the increased fracture risk in patients with COPD. However, on retrospective calculation of fracture risk, the tools identify those patients with COPD at greatest risk of fracture. Identification with a systematic assessment of bone health and addressing prevention and treatment of those at greatest risk of fracture would improve quality of life and outcomes for patients with COPD.

For peer review only

Acknowledgements

With grateful thanks to Prof J Hippisley-Cox and Mr S Hippisley-Cox for use of the QFracture®.

Competing interests

All authors have completed the Unified Competing Interest form (available on request from the corresponding author) and declare: CEB, TMM, JES received an investigator sponsored study grant from Pfizer for the submitted work; CEB reports grants from MRC/Association of British Pharmaceutical Industry (ABPI), TSB, GSK and other support from Chiesi and Boehringer, outside the submitted work; JES reports personal fees from Astra Zeneca, Boehringer-Ingelheim, Nutricia, Chiesi, Sandoz, Novartis, Pfizer, MIMS, RCGP, Cogora and other support from PCRS-UK, Education for Health, Teva and NICE outside the submitted work; no financial relationships with any organisations that might have an interest in the submitted work in the previous three years, no other relationships or activities that could appear to have influenced the submitted work.

Author contributions

CEB, TMM and JES designed study concept and design and are grant holders. RKA conducted the main statistical analysis and wrote the first draft of the manuscript. JEG prepared the THIN data extracts used and assisted with analysis. All authors contributed to the interpretation of the data, writing of the manuscript and critical revisions.

CEB is guarantor.

Ethical approval

The study was approved by an independent Scientific Review Committee (SRC), 16THIN029.

Funding

This study was funded by a COPD "Open Air" research grant from Pfizer.

The funders had no role in study design, data collection and analysis, decision to publish or preparation of the manuscript. CEB and TMM are supported by the NIHR Nottingham BRC. The views expressed are those of the authors and not necessarily those of the NHS, the NIHR or the Department of Health.

Data sharing

No additional data available.

For peer review only

REFERENCES

1 Global Strategy for the Diagnosis Management and Prevention of COPD. Global Initiative for Chronic Obstructive Lung Disease (GOLD) 2017. http://goldcopd.org/gold-2017-global-strategy-diagnosis-management-prevention-copd/ (accessed 13 Apr 2017).

2 Barnes PJ, Celli BR. Systemic manifestations and comorbidities of COPD. *Eur Respir J* 2009;**33**:1165–85. doi:10.1183/09031936.00128008

3 Shane E, Silverberg SJ, Donovan D, *et al.* Osteoporosis in lung transplantation candidates with end-stage pulmonary disease. *Am J Med* 1996;**101**:262–9.

4 Bolton CE, Ionescu AA, Shiels KM, *et al.* Associated Loss of Fat-free Mass and Bone Mineral Density in Chronic Obstructive Pulmonary Disease. *Am J Respir Crit Care Med* 2004;**170**:1286–93. doi:10.1164/rccm.200406-754OC

5 Duckers JM, Evans BA, Fraser WD, *et al.* Low bone mineral density in men with chronic obstructive pulmonary disease. *Respir Res* 2011;**12**:101. doi:10.1186/1465-9921-12-101

6 National Institute for Health and Care Excellence. Osteoporosis: assessing the risk of fragility fracture. 2012.

7 Lehouck A, Boonen S, Decramer M, *et al.* COPD, Bone Metabolism, and Osteoporosis. *Chest* 2011;**139**:648–57. doi:10.1378/chest.10-1427

8 Gjertsen J-E, Baste V, Fevang JM, *et al.* Quality of life following hip fractures: results from the Norwegian hip fracture register. *BMC Musculoskelet Disord* 2016;**17**. doi:10.1186/s12891-016-1111-y

9 Jameson JL, De Groot LJ. *Endocrinology: Adult and Pediatric E-Book*. Elsevier Health Sciences 2015.

10 Peppas NA, Hilt JZ, Thomas JB. *Nanotechnology in Therapeutics: Current Technology and Applications*. Horizon Bioscience 2007.

11 Hakamy A, Bolton CE, Gibson JE, *et al.* Risk of fall in patients with COPD. *Thorax* 2018.

12 Coughlan T, Dockery F. Osteoporosis and fracture risk in older people. *Clin Med* 2014;**14**:187–91. doi:10.7861/clinmedicine.14-2-187

13 Roux C, Wyman A, Hooven FH, *et al.* Burden of non-hip, non-vertebral fractures on quality of life in postmenopausal women. *Osteoporos Int* 2012;**23**:2863–71. doi:10.1007/s00198-012-1935-8

14 Blak BT, Thompson M, Dattani H, *et al.* Generalisability of The Health Improvement Network (THIN) database: demographics, chronic disease prevalence and mortality rates. *Inform Prim Care* 2011;**19**:251–5.

15 Quint JK, Müllerova H, DiSantostefano RL, *et al.* Validation of chronic obstructive pulmonary disease recording in the Clinical Practice Research Datalink (CPRD-GOLD). *BMJ Open* 2014;**4**:e005540. doi:10.1136/bmjopen-2014-005540

16 Scottish Intercollegiate Guidelines Network (SIGN). Management of osteoporosis and the prevention of fragility fractures. Edinburgh: 2015.

For peer review only

17 Lix LM, Quail J, Teare G, *et al.* Performance of comorbidity measures for predicting outcomes in population-based osteoporosis cohorts. *Osteoporos Int* 2011;**22**:2633–43. doi:10.1007/s00198-010-1516-7

18 World Health Organization Collaborating Center for Metabolic Bone Diseases. FRAX® WHO Fracture Risk Assessment Tool. 2011.https://www.sheffield.ac.uk/FRAX/tool.jsp (accessed 18 Sep 2017).

19 ClinRisk Ltd. QFracture-2016® risk calculator. http://www.qfracture.org/ (accessed 20 Sep 2017).

20 Unnanuntana A, Gladnick BP, Donnelly E, *et al.* The assessment of fracture risk. *J Bone Joint Surg Am* 2010;**92**:743–53. doi:10.2106/JBJS.I.00919

21 Miller J, Edwards LD, Agustí A, *et al.* Comorbidity, systemic inflammation and outcomes in the ECLIPSE cohort. *Respir Med* 2013;**107**:1376–84. doi:10.1016/j.rmed.2013.05.001

22 Ferguson GT, Calverley PMA, Anderson JA, *et al.* Prevalence and progression of osteoporosis in patients with COPD: results from the TOwards a Revolution in COPD Health study. *Chest* 2009;**136**:1456–65.

23 National Institute for Health and Care Excellence. Bisphosphonates for treating osteoporosis. 2017.

24 Kanis JA, Johnell O, Oden A, *et al.* The risk and burden of vertebral fractures in Sweden. *Osteoporos Int* 2004;**15**:20–6. doi:10.1007/s00198-003-1463-7

25 Papaioannou A, Morin S, Cheung AM, *et al.* 2010 clinical practice guidelines for the diagnosis and management of osteoporosis in Canada: Summary. CMAJ. 2010;**182**:1864–73. doi:10.1503/cmaj.100771

26 Sibley KM, Voth J, Munce SE, *et al.* Chronic disease and falls in community-dwelling Canadians over 65 years old: a population-based study exploring associations with number and pattern of chronic conditions. *BMC Geriatr* 2014;**14**:22. doi:10.1186/1471-2318-14-22

27 Gonzalez A V., Coulombe J, Ernst P, *et al.* Long-term Use of Inhaled Corticosteroids in COPD and the Risk of Fracture. *Chest* 2018;**153**:321–8. doi:10.1016/J.CHEST.2017.07.002

28 Dennison EM, Compston JE, Flahive J, *et al.* Effect of co-morbidities on fracture risk: findings from the Global Longitudinal Study of Osteoporosis in Women (GLOW). *Bone* 2012;**50**:1288–93. doi:10.1016/j.bone.2012.02.639

29 de Vries F, van Staa TP, Bracke MSGM, *et al.* Severity of obstructive airway disease and risk of osteoporotic fracture. *Eur Respir J* 2005;**25**:879–84. doi:10.1183/09031936.05.00058204

30 National Institute for Health and Care Excellence, NICE. Chronic obstructive pulmonary disease in over 16s: diagnosis and management. NICE 2010.

31 British Thoracic Society, Pulmonary Rehabilitation Guideline, Group. BTS Guideline on Pulmonary Rehabilitation in Adults. *Thorax* 2013;**68**.

32 Dagan N, Cohen-Stavi C, Leventer-Roberts M, *et al.* External validation and comparison of three prediction tools for risk of osteoporotic fractures using data from population based electronic health records: retrospective cohort study. *BMJ* 2017;**356**:i6755. doi:10.1136/BMJ.I6755

For peer review only

33 Tamaki J, Iki M, Kadowaki E, *et al.* Fracture risk prediction using FRAX®: A 10-year follow-up survey of the Japanese Population-Based Osteoporosis (JPOS) Cohort Study. Osteoporos. Int. 2011;**22**:3037–45. doi:10.1007/s00198-011-1537-x

34 Steyerberg EW. *Clinical Prediction Models: A Practical Approach to Development, Validation, and Updating*. Springer 2008. doi:10.1080/10543400903244270

35 Kanis JA, Oden A, Johnell O, *et al.* The use of clinical risk factors enhances the performance of BMD in the prediction of hip and osteoporotic fractures in men and women. *Osteoporos Int* 2007;**18**:1033–46. doi:10.1007/s00198-007-0343-y

36 Hippisley-Cox J, Coupland C. Derivation and validation of updated QFracture algorithm to predict risk of osteoporotic fracture in primary care in the United Kingdom: prospective open cohort study. *BMJ* 2012;**344**:e3427. doi:10.1136/bmj.e3427

37 Cummings SR, Melton LJ. Epidemiology and outcomes of osteoporotic fractures. *Lancet* 2002;**359**:1761–7. doi:10.1016/S0140-6736(02)08657-9

For peer review only

Table 1: Baseline characteristics of patients with COPD and non-COPD subjects

**Descriptor**	**COPD patients**		**Non-COPD subjects**		
	n **=** 80,874	**%**	***n*** **=** 308,999	**%**	***p*-value**
**Mean age at index date (years, SD)**	66.9 (11.0)		66.5 (10.9)		
**Gender**					0.002
Male	42,799	52.9	161,648	52.3	
Female	38,075	47.1	147,351	47.7	
**Follow-up (years, median, IQR)**	5.28	2.6-8.3	5.24	2.6-8.3	
**MRC Dyspnoea Scale** (1 Year either side of diagnosis)					<0.001
1	9,499	11.8	1,168	0.4	
2	19,466	24.1	1,092	0.4	
3	10,488	13.0	446	0.1	
4 & 5	5,237	6.5	177	0.1	
No record	36,184	44.7	306,116	99.1	
**Charlson Comorbidity Index Score**					<0.001
0	0	0.0	172,566	55.9	
1	41,777	51.7	50,955	16.5	
2	13,506	16.7	42,667	13.8	
3	12,694	15.7	23,546	7.6	
≥ 4	12,897	16.0	19,265	6.2	
**Body Mass Index (BMI) (kg/m^2)**					<0.001
Underweight (< 18.5)	3,414	4.2	2,699	0.9	
Normal (18.5 – 24.9)	24,734	30.6	54,267	17.6	
Overweight (25 – 29.9)	23,497	29.1	77,129	25.0	
Obese (≥30)	19,083	23.6	60,280	19.5	
No BMI	10,146	12.6	114,624	37.1	
**Smoking status** (1 Year either side of diagnosis)					<0.001
Never smoked	7,925	9.8	94,800	30.7	
Ex-smoker	38,590	47.7	72,989	23.6	
Current smoker	32,436	40.1	34,691	11.2	
Unknown	1,923	2.4	106,519	34.5	
**History of Falls** (prior to or at diagnosis)					
Personal history	8,969	11.1	26,203	8.5	<0.001
Parental history of fall/osteoporosis	96	0.1	298	0.1	0.076
**Medications** (1 Year either side of diagnosis)					
Oral Glucocorticoid Use	33,618	41.6	19,479	6.3	<0.001
Inhaled Corticosteroid Use	47,574	58.8	21,312	6.9	<0.001

For peer review only

Table 2: Risk of fractures in patients with COPD compared with non-COPD subjects

	Number of fractures	Rate/1,000 person-years	Crude HR (95% CI)	Fully adjusted HR (95% CI)
**Major osteoporotic fractures**				
Non-COPD subjects	6,032	4.32	Reference	Reference
Patients with COPD	2,234	6.64	1.60 (1.52 – 1.69)	1.04 (0.96 – 1.12) [a]
**Hip fracture**				
Non-COPD subjects	3,170	2.26	Reference	Reference
Patients with COPD	1,213	3.57	1.67 (1.56 – 1.80)	1.09 (0.98 – 1.21) [b]

HR – Hazard ratio; CI – Confidence interval

Crude HR – Cox regression model derived HR adjusted for age, sex, and GP practice

[a] Multivariate Cox regression model derived HR was adjusted for age, sex, GP practice, Charlson Comorbidity Index, Body Mass Index, smoking status, inhaled corticosteroid use, antidepressant use and cumulative oral corticosteroid use.

[b] Multivariate Cox regression model derived HR was adjusted for age, sex, GP practice, Charlson Comorbidity Index, Body Mass Index, smoking status, inhaled corticosteroid use and cumulative oral corticosteroid use.

For peer review only

Table 3: Discrimination measures for FRAX® and QFracture® at recommended treatment cut offs for both major osteoporotic and hip fractures

Discriminatory measures	FRAX®	QFracture®
	Measure for ≥ 20% risk	Measure for ≥ 20% risk
**Major Osteoporotic fractures**		
Sensitivity	25.4%	25.2%
Specificity	92.6%	87.7%
Positive Predictive Value	18.8%	12.2%
Negative Predictive Value	94.8%	94.5%
	**Measure for ≥ 3% risk**	**Measure for ≥ 3% risk**
**Hip fracture**		
Sensitivity	78.1%	82.1%
Specificity	60.8%	55.2%
Positive Predictive Value	3.9%	3.6%
Negative Predictive Value	99.3%	99.3%

For peer review only

Figure 1: Kaplan-Meier plots comparing incidence of major osteoporotic fractures at various predicted fracture risk categories in patients with COPD using (a) FRAX® and (b) QFracture®

For peer review only

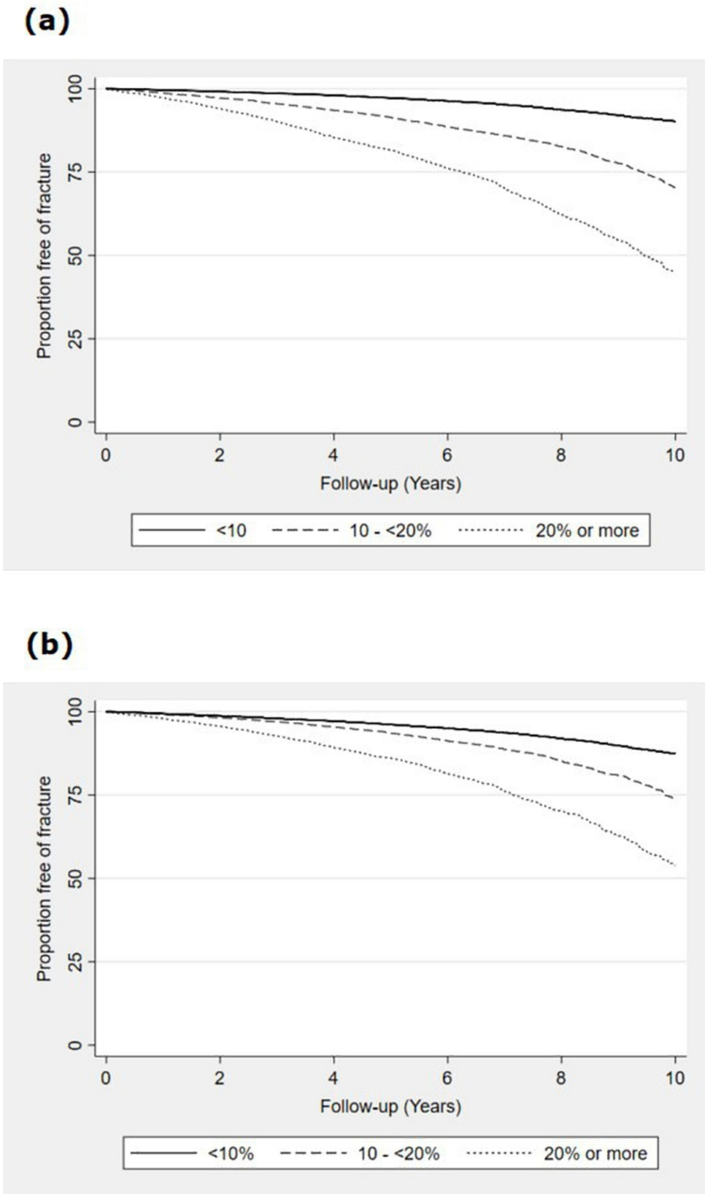

Figure 1: Kaplan-Meier plots comparing incidence of major osteoporotic fractures at various predicted fracture risk categories in patients with COPD using (a) FRAX® and (b) QFracture®

90x153mm (300 x 300 DPI)

Appendix 1

METHODS

Potential confounders

For smoking status, alcohol use, MRC Dyspnoea scale, and a list of prescription drugs, the most recent record within 1 year (before and after) of index date were used. A BMI record within 2 years (before and after) of index date was used.

Where possible BMI was calculated from height and weight records, for patients with a missing BMI record. The BMI was subsequently categorised (underweight: <18.5 kg/m^2, normal: 18.5-<25 kg/m^2, overweight: 25-<30 kg/m^2, obese: >30 kg/m^2).

Having received at least one prescription for inhaled corticosteroids, anti-epileptics, antidepressants, oestrogen-only Hormone Replacement Therapy (HRT) and osteoporosis medications, within 1 year (before and after) of index date were considered as risk factors.

Prediction tools – Input variables

The respective variable definitions as outlined in the algorithms for the prediction tools were used.

Smoking status – In QFracture®, three current smoking categories are provided according to the number of cigarettes smoked daily[1]. To avoid the bias of categorising patients in one of the outlying categories, "current smokers" with no documented number of cigarettes smoked were assigned to the middle category "10-19 cigarettes daily" as done in a recent publication [2]. For FRAX®'s two-category smoking status, former smokers were assigned to the "non-smoker" category as was done in the cohorts used to develop FRAX®.[3]

Alcohol consumption – similarly, for alcohol use in QFracture®, alcohol drinkers with no documented unit/day intake were assigned to "moderate (3-6units/day)".

Missing values for BMI, smoking status, and alcohol use were imputed by multiple imputation using all predictors, resulting in twenty imputed datasets. A complete case sensitivity analysis without imputed variables was also performed.

For peer review only - http://bmjopen.bmj.com/site/about/guidelines.xhtml

References

1 ClinRisk Ltd. QFracture-2016® risk calculator. http://www.qfracture.org/ (accessed 20 Sep 2017).

2 Dagan N, Cohen-Stavi C, Leventer-Roberts M, *et al.* External validation and comparison of three prediction tools for risk of osteoporotic fractures using data from population based electronic health records: retrospective cohort study. *BMJ* 2017;**356**:i6755. doi:10.1136/BMJ.I6755

3 Kanis JA, Oden A, Johnell O, *et al.* The use of clinical risk factors enhances the performance of BMD in the prediction of hip and osteoporotic fractures in men and women. *Osteoporos Int* 2007;**18**:1033–46. doi:10.1007/s00198-007-0343-y

For peer review only

Appendix 2

Table E1: Risk of osteoporosis in patients with COPD compared with non-COPD subjects

Descriptor	Crude HR (95% CI)	Fully adjusted HR (95% CI)
**COPD**		
Non-COPD subjects	Reference	Reference
COPD patients	1.96 (1.87 – 2.06)	1.13 (1.05 – 1.22)
**Charlson Comorbidity Index**		
Score 0	Reference	Reference
Score 1	1.27 (1.18 – 1.36)	1.14 (1.06 – 1.23)
Score 2	1.34 (1.24 – 1.44)	1.27 (1.17 – 1.37)
Score 3	1.41 (1.28 – 1.55)	1.29 (1.17 – 1.42)
Score 4 & more	1.48 (1.33 – 1.64)	1.44 (1.29 – 1.61)
**Body Mass Index** (kg/m^2)		
Underweight (<18.5)	1.93 (1.64 – 2.27)	1.91 (1.63 – 2.25)
Normal (18.5 – 24.9)	Reference	Reference
Overweight (25 – 29.9)	0.64 (0.60 – 0.69)	0.63 (0.58 – 0.67)
Obese (≥ 30)	0.47 (0.43 – 0.51)	0.45 (0.41 – 0.48)
No record	0.50 (0.46 – 0.53)	0.57 (0.52 – 0.61)
**Smoking status**		
Never	Reference	Reference
Ex	1.01 (0.95 – 1.08)	1.02 (0.95 – 1.09)
Current	1.23 (1.13 – 1.33)	1.15 (1.06 – 1.25)
Unknown	0.69 (0.64 – 0.74)	0.77 (0.71 – 0.83)
**Oral Corticosteroid Use**		
Unexposed	Reference	Reference
Exposed	2.79 (2.56 – 3.05)	1.91 (1.73 – 2.10)
**Inhaled Corticosteroid Use**		
No	Reference	Reference
Yes	1.35 (1.26 – 1.45)	1.24 (1.15 – 1.34)

HR – Hazard ratio; CI – Confidence interval

Crude HR – Cox regression model derived HR adjusted for age, sex, and GP practice

The adjusted Hazard Ratio (aHR) was 1.13, 95% CI 1.05 to 1.22, p<0.0001 – the multivariate Cox regression model derived aHR was adjusted for age, sex, GP practice, Charlson comorbidity index, body mass index, smoking status, inhaled corticosteroid use, and cumulative oral corticosteroid use.

For peer review only

Appendix 3

Table E2: Baseline characteristics of patients with COPD aged 40-90 years with no prior diagnosis of osteoporosis or prescription of any anti-resorptive treatment

Descriptor	COPD patients	
	n = 72,559	%
**Mean age at index date (years, SD)**	66.1 (10.7)	
**Gender**		
Female	31,885	43.9
**MRC Dyspnoea Scale** (1 Year either side of diagnosis)		
1	8,882	12.2
2	17,718	24.4
3	9,257	12.8
4 & 5	4,346	6.0
No record	32,356	44.6
**Charlson Comorbidity Index Score**		
0	0	0
1	38,573	53.2
2	11,953	16.5
3	11,110	15.3
≥ 4	10,923	15.1
**Body Mass Index (BMI) (kg/m^2)**		
Underweight (< 18.5)	2,730	3.8
Normal (18.5 – 24.9)	21,791	30.0
Overweight (25 – 29.9)	21,504	29.6
Obese (≥30)	17,627	24.3
No BMI	8,907	12.3
**Smoking status** (1 Year either side of diagnosis)		
Never smoked	7,062	9.7
Ex-smoker	33,810	46.6
Current smoker	29,949	41.3
Unknown	1,738	2.4

For peer review only

STROBE 2007 (v4) Statement—Checklist of items that should be included in reports of *cohort studies*

Section/Topic	Item #	Recommendation	Reported on page #
**Title and abstract**	1	(*a*) Indicate the study's design with a commonly used term in the title or the abstract	1
		(*b*) Provide in the abstract an informative and balanced summary of what was done and what was found	2
**Introduction**			
Background/rationale	2	Explain the scientific background and rationale for the investigation being reported	4
Objectives	3	State specific objectives, including any pre-specified hypotheses	4
**Methods**			
Study design	4	Present key elements of study design early in the paper	5
Setting	5	Describe the setting, locations, and relevant dates, including periods of recruitment, exposure, follow-up, and data collection	5
Participants	6	(*a*) Give the eligibility criteria, and the sources and methods of selection of participants. Describe methods of follow-up	5
		(*b*) For matched studies, give matching criteria and number of exposed and unexposed	5
Variables	7	Clearly define all outcomes, exposures, predictors, potential confounders, and effect modifiers. Give diagnostic criteria, if applicable	5, 6
Data sources/ measurement	8*	For each variable of interest, give sources of data and details of methods of assessment (measurement). Describe comparability of assessment methods if there is more than one group	5, 6
Bias	9	Describe any efforts to address potential sources of bias	6
Study size	10	Explain how the study size was arrived at	-
Quantitative variables	11	Explain how quantitative variables were handled in the analyses. If applicable, describe which groupings were chosen and why	6
Statistical methods	12	(*a*) Describe all statistical methods, including those used to control for confounding	6
		(*b*) Describe any methods used to examine subgroups and interactions	-
		(*c*) Explain how missing data were addressed	6, Appendix 1
		(*d*) If applicable, explain how loss to follow-up was addressed	-
		(*e*) Describe any sensitivity analyses	6
**Results**			

For peer review only

Participants	13*	(a) Report numbers of individuals at each stage of study—eg numbers potentially eligible, examined for eligibility, confirmed eligible, included in the study, completing follow-up, and analysed	8
		(b) Give reasons for non-participation at each stage	-
		(c) Consider use of a flow diagram	-
Descriptive data	14*	(a) Give characteristics of study participants (eg demographic, clinical, social) and information on exposures and potential confounders	8, Table 1 (17)
		(b) Indicate number of participants with missing data for each variable of interest	Table 1 (17)
		(c) Summarise follow-up time (eg, average and total amount)	8
Outcome data	15*	Report numbers of outcome events or summary measures over time	8, 9
Main results	16	(*a*) Give unadjusted estimates and, if applicable, confounder-adjusted estimates and their precision (eg, 95% confidence interval). Make clear which confounders were adjusted for and why they were included	8, 9, Table 2 (18), Appendix 2
		(*b*) Report category boundaries when continuous variables were categorized	Appendix 1
		(*c*) If relevant, consider translating estimates of relative risk into absolute risk for a meaningful time period	-
Other analyses	17	Report other analyses done—eg analyses of subgroups and interactions, and sensitivity analyses	8, 9
**Discussion**			
Key results	18	Summarise key results with reference to study objectives	10
**Limitations**			
Interpretation	20	Give a cautious overall interpretation of results considering objectives, limitations, multiplicity of analyses, results from similar studies, and other relevant evidence	10 - 12
Generalisability	21	Discuss the generalisability (external validity) of the study results	11 & 12
**Other information**			
Funding	22	Give the source of funding and the role of the funders for the present study and, if applicable, for the original study on which the present article is based	13

*Give information separately for cases and controls in case-control studies and, if applicable, for exposed and unexposed groups in cohort and cross-sectional studies.

Note: An Explanation and Elaboration article discusses each checklist item and gives methodological background and published examples of transparent reporting. The STROBE checklist is best used in conjunction with this article (freely available on the Web sites of PLoS Medicine at http://www.plosmedicine.org/, Annals of Internal Medicine at http://www.annals.org/, and Epidemiology at http://www.epidem.com/). Information on the STROBE Initiative is available at www.strobe-statement.org.

For peer review only

Predicting Fracture Risk in Patients with Chronic Obstructive Pulmonary Disease: A UK-based Population-based Cohort Study

Journal:	*BMJ Open*
Manuscript ID	bmjopen-2018-024951.R1
Article Type:	Research
Date Submitted by the Author:	17-Oct-2018
Complete List of Authors:	Akyea, Ralph; University of Nottingham, Nottingham Respiratory Research Unit, NIHR Nottingham Biomedical Research Centre; University of Nottingham, Division of Epidemiology & Public Health<br>McKeever, Tricia; University of Nottingham, Nottingham Respiratory Research Unit, NIHR Nottingham Biomedical Research Centre; University of Nottingham, Division of Epidemiology & Public Health<br>Gibson, Jack; University of Nottingham, Division of Epidemiology & Public Health<br>Scullion, Jane; Institute for Lung Health, University Hospitals of Leicester Glenfield Site<br>Bolton, Charlotte; University of Nottingham, Nottingham Respiratory Research Unit, NIHR Nottingham Biomedical Research Centre
**Primary Subject Heading**:	Respiratory medicine
Secondary Subject Heading:	Epidemiology
Keywords:	Fracture, COPD, fracture risk prediction tool, osteoporosis

SCHOLARONE™
Manuscripts

Predicting Fracture Risk in Patients with Chronic Obstructive Pulmonary Disease: A UK-based Population-based Cohort Study

Ralph K Akyea[1, 2]; Tricia M McKeever[1, 2]; Jack E Gibson[2]; Jane E Scullion[3]; Charlotte E Bolton[1]

Authors affiliations:

[1] Nottingham Respiratory Research Unit, NIHR Nottingham Biomedical Research Centre, School of Medicine, University of Nottingham, UK.

[2] Division of Epidemiology and Public Health, School of Medicine, University of Nottingham, UK.

[3] University Hospitals of Leicester Glenfield Site, Institute for Lung Health, Leicester, UK.

Corresponding author information:

Dr Charlotte E Bolton,

Nottingham Respiratory Research Unit, NIHR Nottingham Biomedical Research Centre, School of Medicine, University of Nottingham, City Hospital Campus, Hucknall Road, Nottingham, NG5 1PB, UK.

Tel: +44 (0)115 8231710

Email: charlotte.bolton@nottingham.ac.uk

Keywords:

Fracture, osteoporosis, COPD, fracture risk prediction tool

Word counts:

Abstract: 283

Main text: 3,012

For peer review only

ABSTRACT

Objectives To assess incidence of hip fracture or major osteoporotic fractures (MOF) in patients with COPD compared to non-COPD subjects and to evaluate the use and performance of fracture risk prediction tools in patients. To assess the prevalence of osteoporosis.

Design A population-based cohort study

Setting UK General Practice health records from The Health Improvement Network database

Participants Patients with an incident COPD diagnosis from 2004-2015 and age, sex and general practice matched non-COPD subjects were studied.

Outcomes Incidence of fracture; accuracy of fracture risk prediction tools in COPD; prevalence and incidence of osteoporosis.

Methods: Cox proportional hazards models were used to assess the incidence rates of fracture and osteoporosis. The discriminatory accuracy (area under the receiver operating curve [ROC]) of fracture risk prediction tools in COPD was assessed.

Results The cohort included 80,874 eligible patients with COPD and 308,999 matched non-COPD subjects. There was an increased risk of fracture in patients with COPD but this was largely mediated through oral corticosteroid use, BMI and smoking. Retrospectively calculated discriminatory accuracies for major osteoporotic fracture were FRAX®: 71.4% (95% CI: 70.6 to 72.2%), QFracture®: 61.4% (95% CI: 60.5 to 62.3%) and for hip fracture both 76.1% (95% CI: 74.9 to 77.2%). Prevalence of coded osteoporosis up to the index date was greater for patients (5.7%) compared to non-COPD subjects (3.9%), $p<0.001$. In those without former osteoporosis, patients (n=73,084) had an increased osteoporosis incidence compared to non-COPD subjects (n=264,544), (adjusted hazard ratio, 1.13, 95% CI 1.05 to 1.22).

For peer review only - http://bmjopen.bmj.com/site/about/guidelines.xhtml

Conclusion COPD patients are at increased risk of fractures and osteoporosis. Despite this, there is no systematic assessment of fracture risk in clinical practice. Fracture risk tools identify those at high-risk of fracture in patients with COPD.

For peer review only

Strengths and limitations of this study

- This study examined electronic health records from a large, nationally representative sample of the UK population.
- A wide range of potential confounders were evaluated and adjusted for in the analyses.
- For the assessment of the fracture prediction tools, the population of patients with COPD used was large, with many fracture events, and included both men and women.
- Data collected in Read codes, in primary care represent only a snap-shot from a clinical consultation.
- The incidence of osteoporosis based on clinical codes, may reflect an underestimation of the true risk of osteoporosis since bone mineral density is not systematically assessed.

For peer review only

INTRODUCTION

Osteoporosis in both male and female patients with COPD is firmly established as one of the core comorbid conditions.[1,2] Over the last decade, it has become clear that osteoporosis is not just an end-stage COPD problem[3] nor just in those on maintenance oral corticosteroids, but it also occurs in a large proportion of those with mild-moderate airflow obstruction and even in steroid naïve patients.[4,5] The Global Initiative for Chronic Obstructive Pulmonary Disease (GOLD strategy recommends that osteoporosis co-existence should be considered in COPD [1] and the UK National Institute for Health and Care Excellence (NICE) Guidelines on osteoporosis considers COPD as a secondary cause of osteoporosis encouraging use of fracture prediction tools.[6]

The causes for osteoporosis in COPD are likely multiple and cumulative, including age, smoking exposure, inactivity, low body mass index (BMI), systemic inflammation and the frequent use of oral corticosteroids.[7] The clinical implications of osteoporosis include increased risk of fractures, poor quality of life, pain and further deterioration in lung function.[8,9] Osteoporosis can also remain undiagnosed as asymptomatic for many years.[10] Fractures are a function of trauma sustained, such as falls which are common in COPD [11], and the quality and architecture of bone. Fractures contribute further pain, poor quality of life, increased mortality and confer a substantial economic burden on health systems, patients and their families.[12,13] Given this, the individual risk of a future fracture in patients with COPD is crucial to determine in patient care and to treat accordingly.

Fracture risk prediction tools based on clinical and personal characteristics have been developed over the years to guide investigation and management of those identified to be at high risk of osteoporotic fractures, worldwide. These include for the UK (and many other regions), FRAX® and QFracture®.[6]

The full extent of fracture risk assessment in patients with COPD is not fully established. The aim of this study was to assess incidence of hip fracture or major osteoporotic fractures (MOF) in patients with COPD compared to non-COPD subjects and to evaluate the use and performance of fracture risk prediction tools in patients. Further, to assess the prevalence of coded osteoporosis up to the time of COPD diagnosis.

For peer review only

For peer review only - http://bmjopen.bmj.com/site/about/guidelines.xhtml

METHODS

Information for this cohort study was obtained from The Health Improvement Network (THIN), an anonymised primary care database representing 6.2% of the total UK population.[14]

The study population consisted of patients 40 years and over with a new Read coded COPD diagnosis during the data collection period 1/1/04-31/12/15, with at least 1 year of record prior to COPD diagnosis.[15] Each patient was matched by age, gender and GP practice to up to four subjects without a history of COPD to generate a matched cohort and assigned the same index date.

Follow up was from the index date to the first record of either the occurrence of the outcome of interest (fracture/osteoporosis), the date of transfer of the patient out of the practice area, death or end of THIN data collection. Read coded diagnoses for osteoporosis *(Appendix 1)* and Read coded hip fracture or major osteoporotic fractures (MOF) (fracture of the hip, proximal humerus, forearm or clinically symptomatic vertebra/spine) were ascertained.

A series of explanatory variables [6,16] determined at baseline (prior to or at index date) included: Charlson Comorbidity Index (CCI) score,[17] Townsend social deprivation score, fall, prior fractures, parental history of fall/osteoporosis, relevant comorbidities and secondary causes of osteoporosis as defined in the FRAX® questionnaire.[18] Records for smoking status, alcohol use, MRC Dyspnoea scale, BMI, and use of specific prescription drugs were restricted to a defined time period.

Individual follow-up time was divided into periods during which participants were considered exposed, or not exposed, to oral corticosteroids (a binary measure). Exposed periods started from prescription date until the first gap of more than 90 days between prescriptions; with individuals considered unexposed from the 91st day onwards. Individuals were considered exposed at study entry if they had received a relevant prescription within 90 days prior. The effect of exposure was assumed to be

For peer review only

constant, and not cumulative, over time (i.e. no time-dependent terms were entered into the model).

Input variables included clinical status, prescription drug use, and demographic characteristics, according to the variables/definitions used in both FRAX® and QFracture® tools,[18,19], additional detail on the method is provided in an online data supplement *(Appendix 2)*. Imputation was used for missing variables.

The 10-year risk score for hip fracture and MOF according to QFracture® (version 2017.0.0.0) and FRAX® for UK without BMD information (desktop version 3.12) were calculated for patients with COPD, aged 40-90 years old. A complete case sensitivity analysis without imputed variables was also performed (Appendix 3).

Statistical analyses

Incidence rates were calculated for both groups using time-dependent Cox proportional hazards regression to estimate hazard ratios (HRs) of osteoporosis and fracture risks, with OCS treated as a time-dependent variable. Confounders were included in the final model when independently changing the HRs for osteoporosis/fracture by at least 5%. A former osteoporosis diagnosis or antiresorptive treatment prior to COPD diagnosis excluded that subject from analyses related to either osteoporosis incidence or risk *(Appendix 4)*. In addition to evaluating incidence in the whole cohort, separate sub-analyses excluded a) patients with COPD and no documented smoking history together with their matched non-COPD subjects and b) those with no prior record of osteoporosis.

To evaluate FRAX® and QFracture®, the outcome was treated as a binary variable (fracture or no fracture). Fracture risk probabilities were categorised based on recommended treatment thresholds (≥ 20% for MOF and ≥3% for hip fracture).[20] To evaluate the overall ability of each tool to discriminate (performance) between those at low and high risks, the area under the receiver operating characteristic (ROC) curve was calculated. Sensitivity, specificity, positive and negative predictive values were calculated. Survival analysis was performed and Kaplan-Meier plots comparing the fracture incidence were generated.

All statistical analyses were performed using Stata 15.0 (StataCorp LP).

For peer review only

Patient involvement

The results and implications of previous research from the team on systematic assessment of osteoporosis in patients with COPD [4] has been discussed extensively in previous patient meetings. Whilst this and other literature has strengthened the GOLD strategy recommendations,[1] evaluation of clinical services would suggest systematic assessment is not done in patients. More recently, patients with COPD out-patient clinics have approached the principal investigator at the time of their "ad hoc osteoporosis" diagnosis to ask why this was not investigated at or closer to COPD diagnosis and how osteoporosis could be assessed. This has led to the development of this grant application with significant patient input in the design and context. The results have been discussed back with representatives on the respiratory research panel. Given the implications for clinical practice, the findings have been discussed extensively at the PPI meeting and a Breathe Easy meeting in early 2018. A lay summary has been developed for the patient newsletter (n>700) and website. In the meantime, members of the respiratory research panel are assisting the PI in planning future work regarding implementation.

For peer review only - http://bmjopen.bmj.com/site/about/guidelines.xhtml

For peer review only

RESULTS

The baseline characteristics are shown in Table 1. A total of 80,874 eligible patients with COPD and 308,999 matched non-COPD subjects were identified. The median follow-up period was 5 years for both patients and non-COPD subjects.

Osteoporosis at index date and incidence

Prevalence of coded osteoporosis up to the index date was greater for patients (5.7%) compared to non-COPD subjects (3.9%), $p<0.001$. Within 1 year (before and after) of the index date, 1,504 (1.86%) patients with COPD had a new recorded diagnosis of osteoporosis compared to 3,059 (1.12%) in matched non-COPD subjects, p <0.001. 3,186 (3.94%) of patients with COPD had a diagnosis of osteoporosis more than a year prior to index date compared to 8,822 (2.86%) for the matched controls p <0.001.

1,457 (1.80%) patients with COPD compared to 3,694 (1.20%) non-COPD subjects, had a record of any diagnostic assessment for osteoporosis, recorded within 1 year (before and after) of the index date, ($p<0.001$).

Demographics remained similar after excluding those with former osteoporosis. Patients with COPD (n=73,084) compared to non-COPD subjects (n=264,544) were significantly more likely to have incident diagnosis of osteoporosis (crude hazard ratio (HR), 1.96; 95% confidence interval [CI] 1.87 to 2.05; *Appendix 5*).

Incidence of Fracture

There was a significantly increased risk of both MOF, crude hazard ratio of 1.60 (95% CI 1.52 to 1.69) and hip fractures: 1.67 (95% CI 1.56 to 1.80) in patients with COPD compared to non-COPD subjects in the unadjusted model. In the fully adjusted models the association were diminished (Table 2). Smoking status altered the effect between COPD and fracture the most, followed by BMI, CCI score and oral corticosteroid.

Sensitivity analysis with participants with no former osteoporosis showed similar results. The risk of MOF was also similar when evaluated in only patients with COPD with a prior history of smoking and their matched controls. However, here, the risk of hip fracture remained significantly increased in the adjusted model compared to non-COPD subjects (aHR, 1.13; 95% CI 1.004 to 1.280; p-value: 0.043).

For peer review only - http://bmjopen.bmj.com/site/about/guidelines.xhtml

Fracture risk prediction tools in COPD

Only 1074 (1.33%) of patients with COPD had a FRAX® assessment READ coded ever documented in the records and 12 patients had a READ coded QFracture® assessment. Within 1 year (before and after) of index date, 248 (0.31%) of patients with COPD had a FRAX® and only 1 patient a QFracture®.

The final population for the discriminatory accuracy analysis comprised 72,559 patients aged 40-90 years with COPD and no prior diagnosis of osteoporosis or prescription of any anti-resorptive treatment (*demographics in Appendix 6*). This included 4,605 (6.4%) who experienced a MOF and 1,444 (2.0%) who experienced hip fracture.

When the FRAX® and QFracture® scores were calculated for patients with COPD, for hip fracture 29,035 (40.0%) had a risk ≥3% using FRAX® and 33,065 (45.6%) using the QFracture®. For MOF, 6,221 (8.6%) of patients had a risk ≥20% using FRAX® and 9,546 (13.2%) using QFracture®.

Both risk tools had a similar discriminatory accuracy for hip fracture (FRAX® 76.1%, 95% CI 74.9 to 77.2% and QFracture® 76.1%, 95% CI 74.9 to 77.2%). FRAX®, however, had a higher accuracy for MOF (71.4% 95% CI 70.6% to 72.2%) than QFracture® (61.4% 95% CI 60.5% to 62.3%).

The discriminatory accuracies were better in women than men. The performance of the prediction tools was similar in the patients aged 50-90 years compared to 40-90-year olds. Table 3 shows the results for the sensitivity, specificity, positive and negative predictive values assessed for the performance of the prediction tools at ≥3% risk probability for hip fracture and ≥20% risk probability for major osteoporotic fractures. At a 20% fracture risk cut-off for MOF, FRAX® identified 25.4% (95% CI, 22.7% to 28.1%) (sensitivity) of those who went on to experience an MOF, QFracture® was 25.2% (95% CI, 22.5% to 27.9%). The specificity, positive predictive value (PPV) and negative predictive value (NPV) were 92.6% (95% CI, 91.0 to 94.2), 18.8% (95% CI, 16.4% to 21.1%) and 94.8% (95% CI, 93.4% to 96.2%) for FRAX® and 87.7% (95% CI, 85.7% to 89.7%), 12.2% (95% CI, 10.2% to 14.2%) and 94.5% (95% CI, 93.1% to 95.9%) respectively for QFracture®. At a 3% risk cut-off for hip fractures, FRAX® sensitivity, specificity, PPV and NPV were 78.1% (95% CI, 75.6% to 80.7%), 60.8%

For peer review only - http://bmjopen.bmj.com/site/about/guidelines.xhtml

(95% CI, 57.8% to 63.8%), 3.9% (95% CI, 2.7% to 5.1%), 99.3% (95% CI, 98.8% to 99.8%) respectively and 82.1% (95% CI, 79.7% to 84.5%), 55.2% (95% CI, 52.1% to 58.3%), 3.6% (95% CI, 2.5% to 4.8%) and 99.3% (95% CI, 98.8% to 99.8%) respectively for QFracture®.

The Kaplan-Meier plots for time to first MOF for QFracture® and FRAX® are presented in Figure 1.

For peer review only

For peer review only - http://bmjopen.bmj.com/site/about/guidelines.xhtml

DISCUSSION

Using UK primary care electronic health records, we have reported on the burden of fractures in patients with COPD with both hip and MOF increased in patients with COPD compared to age, gender and GP surgery matched subjects. Despite the increased fracture risk and recommendations in the NICE osteoporosis guidelines, fracture risk prediction tools are rarely coded. However, where the risk score was retrospectively calculated, the risk prediction tools identify those at risk of hip fracture or MOF. Therefore, fracture risk prediction and subsequent targeted therapy and management could transform multi-morbidity management of COPD. In addition, we report that the prevalence and incidence of osteoporosis, a risk for fracture, in patients with COPD, is far greater than in non-COPD subjects.

Prevalence of osteoporosis varies widely in the different studies of patients with COPD. This is mainly dependent on the severity of COPD,[4,5] whether osteoporosis was systematically sought or self-reported [4,21], and whether patients included were on oral corticosteroids.[3] A prevalence of 23-32% has been reported where BMD was systematically performed [22].[4], while 14% of patients with COPD self-reported osteoporosis compared to 5% in those without COPD.[21] The prevalence of coded osteoporosis in the GP health records was, however, far lower at 5.7% than the reported prevalence from clinical studies when osteoporosis and BMD are systematically assessed. This raises the question of subclinical, undiagnosed disease leading to a missed opportunity for intervention and strengthening the need for a systematic assessment especially when cost-efficient anti-resorptive treatment is available.[23]

There is growing consensus on COPD being a secondary cause of osteoporosis, including within the NICE clinical guideline on osteoporosis where fracture risk prediction tools are recommended, yet in practice seem rarely done.[6] Whilst osteoporosis in itself leads to pain and poor quality of life,[24] ultimately osteoporosis treatment aims to reduce the risk of fracture.[23,25] Risk factors for fracture include osteoporosis but also falls, which, are greater in patients with COPD.[11,26] Whilst the increased risk of fractures in COPD has previously been considered,[27] they have not assessed incidence from time of COPD diagnosis or only reported as part of a larger study of post-menopausal women [28] or analysed the history of obstructive airway disease (both COPD and

For peer review only - http://bmjopen.bmj.com/site/about/guidelines.xhtml

asthma together) before the index date of osteoporotic fracture in both cases and controls over the age of 18 years.[29]

Little is known about the use of fracture risk assessment tools in patients with COPD. A number of validation studies have performed independent assessments to predict subsequent fracture in the general population.[30,31] The studies differ widely in sample size, methodology, and techniques used to assess performance.[32] Discrimination for FRAX® (without BMD incorporation) and QFracture® have both been reported as good.[30,33,34] The results from this COPD study are comparable to the general population validation studies,[30,33,34] however, the AUC for MOF using QFracture® was lower than that reported in other studies. The discrimination from our study was better in women and for hip fracture as it is in the general population studies – both associated with the greatest morbidity and mortality.[35] The discrimination appeared similar within the 40-90 and 50-90 year-old groups. Despite the two tools having differences in their approach to calculating fracture risks, both predict fractures satisfactorily in patients with COPD and will thus be helpful in selecting high-risk patients. Available fracture prevention therapy (anti-resorptive agents) are very effective, safely yielding 40-60% reduction in the risk of fracture.[25] These medications are cost-effective in high-risk patients –reduces morbidity, mortality and health care cost associated with osteoporotic fractures.[23] The fracture prediction tools could be integrated into COPD annual assessments or at COPD diagnosis. Identification of patients at high risk is valuable information to guide and optimise treatment options. Though the optimal pathways for this integration is required.

The use of oral corticosteroids has been considered to be a major contributory factor in the development of osteoporosis. However, osteoporosis has been reported in patients with no oral corticosteroid use.[4,5] Other known osteoporosis risk factors are also likely to contribute in patients with COPD including smoking, a low BMI, physical inactivity and systemic inflammation. Some of these risk factors could be moderated through education, smoking cessation, pulmonary rehabilitation and lifestyle changes.[36,37] Recognition of the scale and impact of fracture risk draws further necessary attention to these interventions to aim to prevent and reduce risks, alongside appropriate pharmacotherapy.

For peer review only

The study had several strengths in its methods, analyses, findings, and implications for clinical practice. Firstly, this research was population-based and compared patients with COPD with age-sex matched control subjects from the same general practice. Its external validity and hence generalisability was high because THIN database is representative of the UK population.[14] There was a substantial duration of follow-up. A wide range of potential confounders were also evaluated and adjusted for in the analyses.

For the assessment of the fracture prediction tools, the population of patients with COPD used was large, with many fracture events, and included both men and women. This minimised the likelihood of a selection bias. The assessments of the prediction tools were done using the same population, therefore minimising the effect of confounding for a difference in performance. We are presently not aware of studies that have determined the performance of the recommended fracture prediction tools in the sub-population of patients with COPD. The dataset was using UK electronic health records but is likely representative of other countries in representing the scale of the problem and the utility of the risk prediction scores.

Regarding limitations, some variables might be subject to information or reporting bias, including patient reported alcohol intake, use of cigarettes or their awareness of relevant family history. The possibility of residual confounding can also not be excluded as risk factors such as physical activity, diet and ethnicity could not be adjusted for in the analyses. An accepted definition of fractures types was used; however, it is difficult to determine the cause of fracture based simply on fracture site, with no additional information. Unlike studies which assess BMD systematically, this is not currently done in clinical practice, nor are the fracture risk scores routinely calculated as highlighted here. Therefore, the incidence of osteoporosis based on clinical codes likely reflects an underestimation of the true increased incidence/risk of osteoporosis.

In summary, despite validated fracture risk prediction tools, there was very little assessment of the increased fracture risk in patients with COPD. However, on retrospective calculation of fracture risk, the tools identify those patients with COPD at greatest risk of fracture. Identification with a systematic assessment of bone health and

For peer review only - http://bmjopen.bmj.com/site/about/guidelines.xhtml

addressing prevention and treatment of those at greatest risk of fracture has the potential to improve outcomes for patients with COPD.

For peer review only

For peer review only - http://bmjopen.bmj.com/site/about/guidelines.xhtml

Acknowledgements

With grateful thanks to Prof J Hippisley-Cox and Mr S Hippisley-Cox for use of the QFracture®.

Competing interests

All authors have completed the Unified Competing Interest form (available on request from the corresponding author) and declare: CEB, TMM, JES received an investigator sponsored study grant from Pfizer for the submitted work; CEB reports grants from MRC/Association of British Pharmaceutical Industry (ABPI), TSB, GSK and other support from Chiesi and Boehringer, outside the submitted work; JES reports personal fees from Astra Zeneca, Boehringer-Ingelheim, Nutricia, Chiesi, Sandoz, Novartis, Pfizer, MIMS, RCGP, Cogora and other support from PCRS-UK, Education for Health, Teva and NICE outside the submitted work; no financial relationships with any organisations that might have an interest in the submitted work in the previous three years, no other relationships or activities that could appear to have influenced the submitted work.

Author contributions

CEB, TMM and JES designed study concept and design and are grant holders. RKA conducted the main statistical analysis and wrote the first draft of the manuscript. JEG prepared the THIN data extracts used and assisted with analysis. All authors contributed to the interpretation of the data, writing of the manuscript and critical revisions.

CEB is guarantor.

Ethical approval

The study was approved by an independent Scientific Review Committee (SRC), 16THIN029.

Funding

This study was funded by a COPD "Open Air" research grant from Pfizer.

The funders had no role in study design, data collection and analysis, decision to publish or preparation of the manuscript. CEB and TMM are supported by the NIHR Nottingham BRC. The views expressed are those of the authors and not necessarily those of the NHS, the NIHR or the Department of Health.

Data sharing

For peer review only

No additional data available.

For peer review only

REFERENCES

1 Global Strategy for the Diagnosis Management and Prevention of COPD. Global Initiative for Chronic Obstructive Lung Disease (GOLD) 2017. http://goldcopd.org/gold-2017-global-strategy-diagnosis-management-prevention-copd/ (accessed 13 Apr 2017).

2 Barnes PJ, Celli BR. Systemic manifestations and comorbidities of COPD. *Eur Respir J* 2009;**33**:1165–85. doi:10.1183/09031936.00128008

3 Shane E, Silverberg SJ, Donovan D, *et al.* Osteoporosis in lung transplantation candidates with end-stage pulmonary disease. *Am J Med* 1996;**101**:262–9.

4 Bolton CE, Ionescu AA, Shiels KM, *et al.* Associated Loss of Fat-free Mass and Bone Mineral Density in Chronic Obstructive Pulmonary Disease. *Am J Respir Crit Care Med* 2004;**170**:1286–93. doi:10.1164/rccm.200406-754OC

5 Duckers JM, Evans BA, Fraser WD, *et al.* Low bone mineral density in men with chronic obstructive pulmonary disease. *Respir Res* 2011;**12**:101. doi:10.1186/1465-9921-12-101

6 National Institute for Health and Care Excellence. Osteoporosis: assessing the risk of fragility fracture. 2012.

7 Lehouck A, Boonen S, Decramer M, *et al.* COPD, Bone Metabolism, and Osteoporosis. *Chest* 2011;**139**:648–57. doi:10.1378/chest.10-1427

8 Gjertsen J-E, Baste V, Fevang JM, *et al.* Quality of life following hip fractures: results from the Norwegian hip fracture register. *BMC Musculoskelet Disord* 2016;**17**. doi:10.1186/s12891-016-1111-y

9 Jameson JL, De Groot LJ. *Endocrinology: Adult and Pediatric E-Book*. Elsevier Health Sciences 2015.

10 Peppas NA, Hilt JZ, Thomas JB. *Nanotechnology in Therapeutics: Current Technology and Applications*. Horizon Bioscience 2007.

11 Hakamy A, Bolton CE, Gibson JE, *et al.* Risk of fall in patients with COPD. *Thorax* 2018.

12 Coughlan T, Dockery F. Osteoporosis and fracture risk in older people. *Clin Med* 2014;**14**:187–91. doi:10.7861/clinmedicine.14-2-187

13 Roux C, Wyman A, Hooven FH, *et al.* Burden of non-hip, non-vertebral fractures on quality of life in postmenopausal women. *Osteoporos Int* 2012;**23**:2863–71. doi:10.1007/s00198-012-1935-8

14 Blak BT, Thompson M, Dattani H, *et al.* Generalisability of The Health Improvement Network (THIN) database: demographics, chronic disease prevalence and mortality rates. *Inform Prim Care* 2011;**19**:251–5.

15 Quint JK, Müllerova H, DiSantostefano RL, *et al.* Validation of chronic obstructive pulmonary disease recording in the Clinical Practice Research Datalink (CPRD-GOLD). *BMJ Open* 2014;**4**:e005540. doi:10.1136/bmjopen-2014-005540

16 Scottish Intercollegiate Guidelines Network (SIGN). Management of osteoporosis and the prevention of fragility fractures. Edinburgh: 2015.

17 Lix LM, Quail J, Teare G, *et al.* Performance of comorbidity measures for predicting

For peer review only

For peer review only - http://bmjopen.bmj.com/site/about/guidelines.xhtml

outcomes in population-based osteoporosis cohorts. *Osteoporos Int* 2011;**22**:2633–43. doi:10.1007/s00198-010-1516-7

18 World Health Organization Collaborating Center for Metabolic Bone Diseases. FRAX® WHO Fracture Risk Assessment Tool. 2011.https://www.sheffield.ac.uk/FRAX/tool.jsp (accessed 18 Sep 2017).

19 ClinRisk Ltd. QFracture-2016® risk calculator. http://www.qfracture.org/ (accessed 20 Sep 2017).

20 Unnanuntana A, Gladnick BP, Donnelly E, *et al.* The Assessment of Fracture Risk. *J Bone Jt Surgery-American Vol* 2010;**92**:743–53. doi:10.2106/JBJS.I.00919

21 Miller J, Edwards LD, Agustí A, *et al.* Comorbidity, systemic inflammation and outcomes in the ECLIPSE cohort. *Respir Med* 2013;**107**:1376–84. doi:10.1016/j.rmed.2013.05.001

22 Ferguson GT, Calverley PMA, Anderson JA, *et al.* Prevalence and progression of osteoporosis in patients with COPD: results from the TOwards a Revolution in COPD Health study. *Chest* 2009;**136**:1456–65.

23 National Institute for Health and Care Excellence. Bisphosphonates for treating osteoporosis. 2017.

24 Kanis JA, Johnell O, Oden A, *et al.* The risk and burden of vertebral fractures in Sweden. *Osteoporos Int* 2004;**15**:20–6. doi:10.1007/s00198-003-1463-7

25 Papaioannou A, Morin S, Cheung AM, *et al.* 2010 clinical practice guidelines for the diagnosis and management of osteoporosis in Canada: Summary. CMAJ. 2010;**182**:1864–73. doi:10.1503/cmaj.100771

26 Sibley KM, Voth J, Munce SE, *et al.* Chronic disease and falls in community-dwelling Canadians over 65 years old: a population-based study exploring associations with number and pattern of chronic conditions. *BMC Geriatr* 2014;**14**:22. doi:10.1186/1471-2318-14-22

27 Gonzalez A V., Coulombe J, Ernst P, *et al.* Long-term Use of Inhaled Corticosteroids in COPD and the Risk of Fracture. *Chest* 2018;**153**:321–8. doi:10.1016/J.CHEST.2017.07.002

28 Dennison EM, Compston JE, Flahive J, *et al.* Effect of co-morbidities on fracture risk: findings from the Global Longitudinal Study of Osteoporosis in Women (GLOW). *Bone* 2012;**50**:1288–93. doi:10.1016/j.bone.2012.02.639

29 de Vries F, van Staa TP, Bracke MSGM, *et al.* Severity of obstructive airway disease and risk of osteoporotic fracture. *Eur Respir J* 2005;**25**:879–84. doi:10.1183/09031936.05.00058204

30 Dagan N, Cohen-Stavi C, Leventer-Roberts M, *et al.* External validation and comparison of three prediction tools for risk of osteoporotic fractures using data from population based electronic health records: retrospective cohort study. *BMJ* 2017;**356**:i6755. doi:10.1136/BMJ.I6755

31 Tamaki J, Iki M, Kadowaki E, *et al.* Fracture risk prediction using FRAX®: A 10-year follow-up survey of the Japanese Population-Based Osteoporosis (JPOS) Cohort Study. Osteoporos. Int. 2011;**22**:3037–45. doi:10.1007/s00198-011-1537-x

32 Steyerberg EW. *Clinical Prediction Models: A Practical Approach to Development,*

For peer review only

Validation, and Updating. Springer 2008. doi:10.1080/10543400903244270

33 Kanis JA, Oden A, Johnell O, *et al.* The use of clinical risk factors enhances the performance of BMD in the prediction of hip and osteoporotic fractures in men and women. *Osteoporos Int* 2007;**18**:1033–46. doi:10.1007/s00198-007-0343-y

34 Hippisley-Cox J, Coupland C. Derivation and validation of updated QFracture algorithm to predict risk of osteoporotic fracture in primary care in the United Kingdom: prospective open cohort study. *BMJ* 2012;**344**:e3427. doi:10.1136/bmj.e3427

35 Cummings SR, Melton LJ. Epidemiology and outcomes of osteoporotic fractures. *Lancet* 2002;**359**:1761–7. doi:10.1016/S0140-6736(02)08657-9

36 National Institute for Health and Care Excellence, NICE. Chronic obstructive pulmonary disease in over 16s: diagnosis and management. NICE 2010.

37 British Thoracic Society, Pulmonary Rehabilitation Guideline, Group. BTS Guideline on Pulmonary Rehabilitation in Adults. *Thorax* 2013;**68**.

For peer review only

Table 1: Baseline characteristics of patients with COPD and non-COPD subjects

Descriptor	COPD patients		Non-COPD subjects		
	n = 80,874	%	*n* = 308,999	%	*p*-value
**Mean age at index date (years, SD)**	66.9 (11.0)		66.5 (10.9)		
**Gender**					0.002
Male	42,799	52.9	161,648	52.3	
Female	38,075	47.1	147,351	47.7	
**Follow-up (years, median, IQR)**	5.28	2.6-8.3	5.24	2.6-8.3	
**MRC Dyspnoea Scale** (1 Year either side of diagnosis)					<0.001
1	9,499	11.8	1,168	0.4	
2	19,466	24.1	1,092	0.4	
3	10,488	13.0	446	0.1	
4 & 5	5,237	6.5	177	0.1	
No record	36,184	44.7	306,116	99.1	
**Charlson Comorbidity Index Score**					<0.001
0	0	0.0	172,566	55.9	
1	41,777	51.7	50,955	16.5	
2	13,506	16.7	42,667	13.8	
3	12,694	15.7	23,546	7.6	
≥ 4	12,897	16.0	19,265	6.2	
**Body Mass Index (BMI) (kg/m²)**					<0.001
Underweight (< 18.5)	3,414	4.2	2,699	0.9	
Normal (18.5 – 24.9)	24,734	30.6	54,267	17.6	
Overweight (25 – 29.9)	23,497	29.1	77,129	25.0	
Obese (≥30)	19,083	23.6	60,280	19.5	
No BMI	10,146	12.6	114,624	37.1	
**Smoking status** (1 Year either side of diagnosis)					<0.001
Never smoked	7,925	9.8	94,800	30.7	
Ex-smoker	38,590	47.7	72,989	23.6	
Current smoker	32,436	40.1	34,691	11.2	
Unknown	1,923	2.4	106,519	34.5	
**History of Falls** (prior to or at diagnosis)					
Personal history	8,969	11.1	26,203	8.5	<0.001
Parental history of fall/osteoporosis	96	0.1	298	0.1	0.076
**Medications** (1 Year either side of diagnosis)					
Oral Glucocorticoid Use	33,618	41.6	19,479	6.3	<0.001
Inhaled Corticosteroid Use	47,574	58.8	21,312	6.9	<0.001

For peer review only

For peer review only

For peer review only - http://bmjopen.bmj.com/site/about/guidelines.xhtml

Table 2: Risk of fractures in patients with COPD compared with non-COPD subjects

	Number of fractures	Rate/1,000 person-years	Crude HR (95% CI)	Fully adjusted HR (95% CI)
**Major osteoporotic fractures**				
Non-COPD subjects	6,032	4.32 (4.22 – 4.44)	Reference	Reference
Patients with COPD	2,234	6.64 (6.37 – 6.92)	1.60 (1.52 – 1.69)	1.04 (0.96 – 1.12) [a]
**Hip fracture**				
Non-COPD subjects	3,170	2.26 (2.18 – 2.34)	Reference	Reference
Patients with COPD	1,213	3.57 (3.38 – 3.78)	1.67 (1.56 – 1.80)	1.09 (0.98 – 1.21) [b]

HR – Hazard ratio; CI – Confidence interval

Crude HR – Cox regression model derived HR adjusted for age, sex, and GP practice

[a] Multivariate Cox regression model derived HR was adjusted for age, sex, GP practice, Charlson Comorbidity Index, Body Mass Index, smoking status, inhaled corticosteroid use, antidepressant use and cumulative oral corticosteroid use.

[b] Multivariate Cox regression model derived HR was adjusted for age, sex, GP practice, Charlson Comorbidity Index, Body Mass Index, smoking status, inhaled corticosteroid use and cumulative oral corticosteroid use.

For peer review only

Table 3: Discrimination measures for FRAX® and QFracture® at recommended treatment cut offs for both major osteoporotic and hip fractures

Discriminatory measures	FRAX®	QFracture®
	**Measure for ≥ 20% risk** (95% CI)	**Measure for ≥ 20% risk** (95% CI)
**Major Osteoporotic fractures**		
Sensitivity	25.4% (22.7-28.1%)	25.2% (22.5-27.9%)
Specificity	92.6% (91.0-94.2%)	87.7% (85.7-89.7%)
Positive Predictive Value	18.8% (16.4-21.1%)	12.2% (10.2-14.2%)
Negative Predictive Value	94.8% (93.4-96.2%)	94.5% (93.1-95.9%)
	**Measure for ≥ 3% risk**	**Measure for ≥ 3% risk**
**Hip fracture**		
Sensitivity	78.1% (75.6-80.7%)	82.1% (79.7-84.5%)
Specificity	60.8% (57.8-63.8%)	55.2% (52.1-58.3%)
Positive Predictive Value	3.9% (2.7-5.1%)	3.6% (2.5-4.8%)
Negative Predictive Value	99.3% (98.8-99.8%)	99.3% (98.8-99.8%)

CI – Confidence interval

For peer review only

Figure 1: Kaplan-Meier plots comparing incidence of major osteoporotic fractures at various predicted fracture risk categories in patients with COPD using (a) FRAX® and (b) QFracture®

For peer review only

(a)

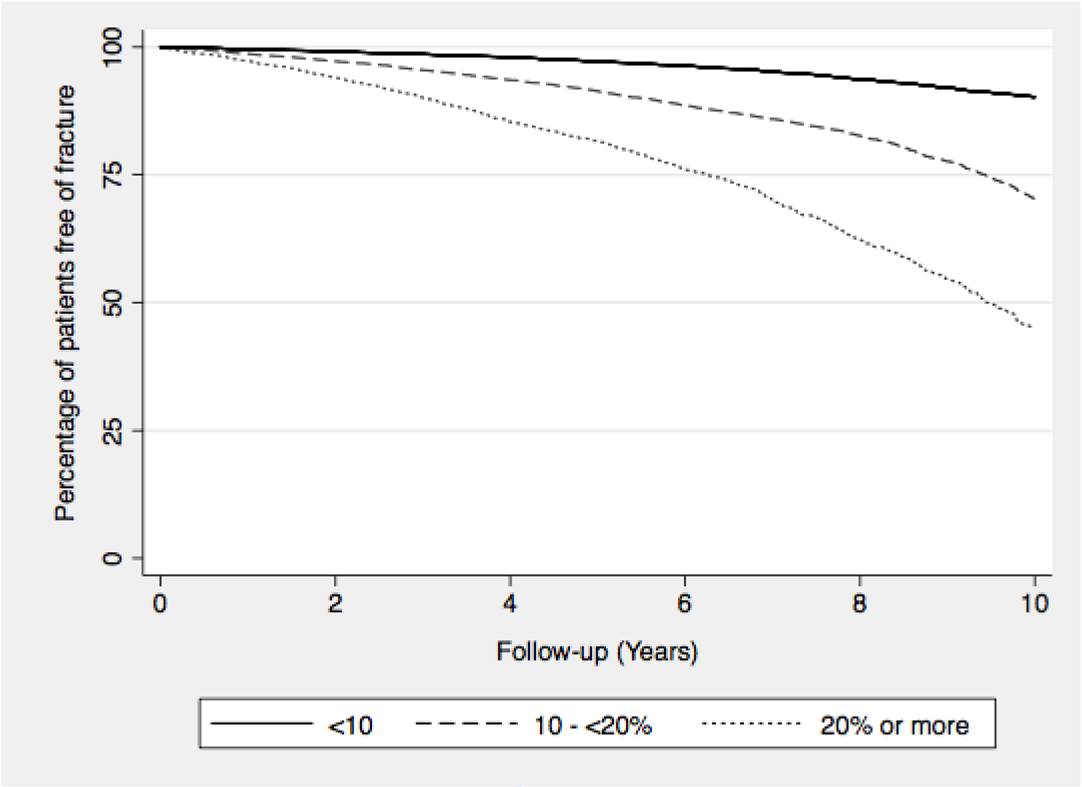
Percentage of patients free of fracture
0
25
50
75
100
0
2
4
6
8
10
Follow-up (Years)
<10
10 - <20%
20% or more

(b)

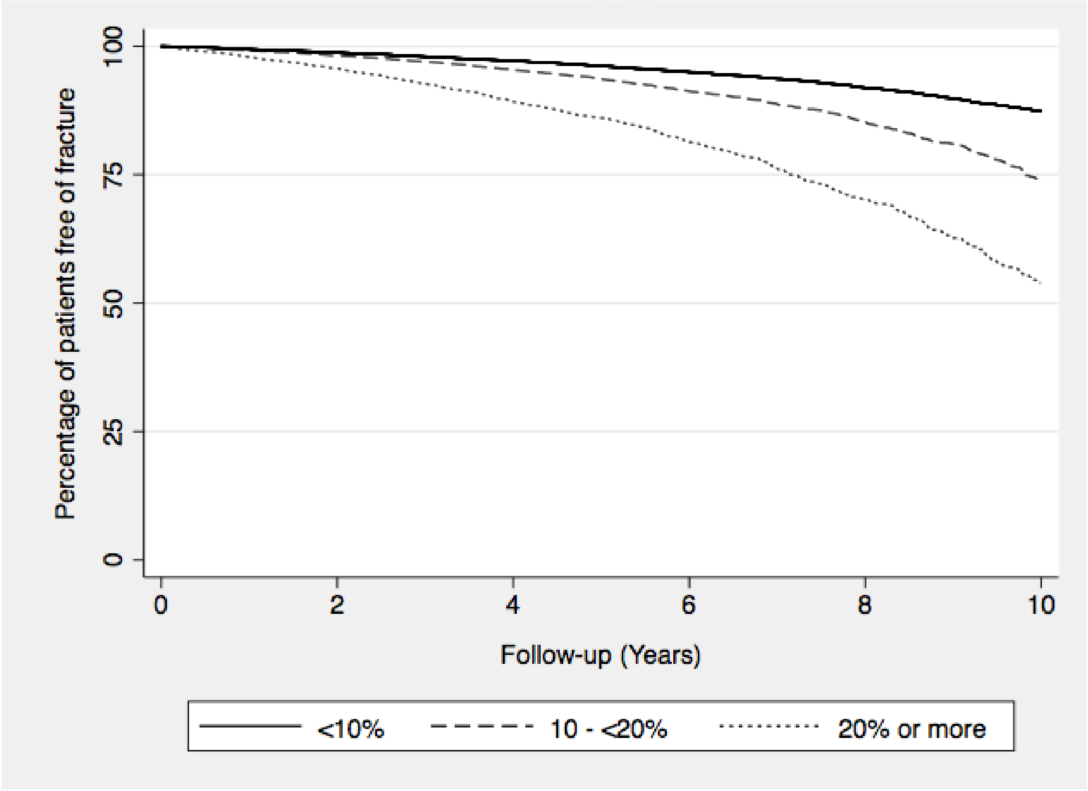
Percentage of patients free of fracture
0
25
50
75
100
0
2
4
6
8
10
Follow-up (Years)
<10%
10 - <20%
20% or more

Appendix 1

Read code definitions for selected input variables

Variable	Read codes
COPD	H3...00, H3...11, H31..00, H310.00, H310000, H310z00, H311.00, H311000, H311100, H311z00, H312.00, H312000, H312011, H312100, H312300, H312z00, H313.00, H31y.00, H31y100, H31yz00, H31z.00, H32..00, H320.00, H320000, H320100, H320200, H320300, H320311, H320z00, H321.00, H322.00, H32y.00, H32y000, H32y100, H32y111, H32y200, H32yz00, H32z.00, H36..00, H37..00, H38..00, H39..00, H3A..00, H3y..00, H3y..11, H3z..00, H3z..11
Osteoporosis	585O.00, 58E4.00, 58E8.00, 58EA.00, 58EE.00, 58EG.00, 58EK.00, 58EM.00, 58ES.00, 58EV.00, 7230A, 7230B, 7230D, 7230PM, 7230PT, N330.00, N330000, N330100, N330200, N330300, N330400, N330500, N330600, N330700, N330800, N330900, N330A00, N330B00, N330C00, N330D00, N330z00, N331200, N331300, N331400, N331500, N331600, N331800, N331900, N331A00, N331B00, N331M00, N331N00, NyuB000, NyuB100, NyuB200, NyuB800
Antiresorptive treatment *(drug code)*	97138998, 99158998, 99158997, 97139998, 96920998, 96789998, 93478998, 97140998, 97218998, 93975992, 83457998, 97064992, 83456998, 96897998, 96020992, 96901998, 95879992, 98249990, 97031992, 98581990, 99018990, 98198990, 62945979, 96737998, 97066992, 97051992, 97780990, 98199990, 61594979, 99261990, 96604992, 92004979, 97248990, 99263990, 94089992, 93127992, 94756992, 91526998, 89828998, 88144998, 88144997, 88225998, 89434998, 93502998, 99862998, 95304998, 93228997, 96904998, 93228998, 95304996, 99862997, 95304997, 93228996, 99864998, 91997998, 91998998, 87933998, 81073998, 61612979, 87155998, 87154998, 88542998, 91378998, 82066998,

For peer review only

Antiresorptive treatment *(drug code)*	82065998, 81256998, 81255998, 91190996, 89518998, 91190998, 91191998, 86599998, 91190997, 91191997, 93692990, 81472998, 94276990, 93827990, 92431990, 94161990, 93610990, 94245990, 61524979, 99883979, 93828990, 99867979, 95572998, 99758998, 96764998, 97398992, 95244990, 89367998, 86562998, 86561998, 87645998, 87644998, 86079998, 86076998, 91533998, 87151998, 81270998, 91027998, 93617996, 93618996, 93618997, 93617997, 90527998, 86566998, 91028998, 87137998, 87136998, 91674998, 86564998, 86567998, 87135998, 93089979, 99357998, 84212998, 84691998, 89021998, 91764998, 90551998, 91763998, 81869998, 91764997, 91763997, 89354979, 92813997, 93402998, 92813998, 98527996, 93403996, 98527998, 93403998, 93402996, 84531998, 58602979, 87606998, 85936998, 81112998, 97865998, 85935998, 81111998, 76983978, 83078978

For peer review only

Appendix 2

METHODS

Potential confounders

For smoking status, alcohol use, MRC Dyspnoea scale, and a list of prescription drugs, the most recent record within 1 year (before and after) of index date were used. A BMI record within 2 years (before and after) of index date was used.

Where possible BMI was calculated from height and weight records, for patients with a missing BMI record. The BMI was subsequently categorised (underweight: <18.5 kg/m^2, normal: 18.5-<25 kg/m^2, overweight: 25-<30 kg/m^2, obese: >30 kg/m^2).

Having received at least one prescription for inhaled corticosteroids, anti-epileptics, antidepressants, oestrogen-only Hormone Replacement Therapy (HRT) and osteoporosis medications, within 1 year (before and after) of index date were considered as risk factors.

Prediction tools – Input variables

The respective variable definitions as outlined in the algorithms for the prediction tools were used.

Smoking status – In QFracture®, three current smoking categories are provided according to the number of cigarettes smoked daily[1]. To avoid the bias of categorising patients in one of the outlying categories, "current smokers" with no documented number of cigarettes smoked were assigned to the middle category "10-19 cigarettes daily" as done in a recent publication [2]. For FRAX®'s two-category smoking status, former smokers were assigned to the "non-smoker" category as was done in the cohorts used to develop FRAX®.[3]

Alcohol consumption – similarly, for alcohol use in QFracture®, alcohol drinkers with no documented unit/day intake were assigned to "moderate (3-6units/day)".

Missing values for BMI, smoking status, and alcohol use were imputed by multiple imputation using all predictors, resulting in twenty imputed datasets.[4] A complete case sensitivity analysis without imputed variables was also performed (Appendix 3).

For peer review only - http://bmjopen.bmj.com/site/about/guidelines.xhtml

References

1 ClinRisk Ltd. QFracture-2016® risk calculator. http://www.qfracture.org/ (accessed 20 Sep 2017).

2 Dagan N, Cohen-Stavi C, Leventer-Roberts M, *et al.* External validation and comparison of three prediction tools for risk of osteoporotic fractures using data from population based electronic health records: retrospective cohort study. *BMJ* 2017;**356**:i6755. doi:10.1136/BMJ.I6755

3 Kanis JA, Oden A, Johnell O, *et al.* The use of clinical risk factors enhances the performance of BMD in the prediction of hip and osteoporotic fractures in men and women. *Osteoporos Int* 2007;**18**:1033–46. doi:10.1007/s00198-007-0343-y

4 Horton NJ, Lipsitz SR. Multiple Imputation in Practice. *Am Stat* 2001;**55**:244–54. doi:10.1198/000313001317098266

For peer review only

Appendix 3

Fracture risk prediction tools in COPD (Complete case analysis)

Of the 72,559 patients aged 40-90 years with COPD and no prior diagnosis of osteoporosis or prescription of any anti-resorptive treatment, 41,879 (57.7%) of patients had complete data. Amongst the patients with complete data, 2,649 (6.3%) experienced a MOF and 806 (1.9%) experienced hip fracture.

Both risk tools had about the same discriminatory accuracy as that obtained from the entire cohort with imputed data. The AUC for hip fracture was 75.6%, 95% CI 74.0% to 77.1% for FRAX® and 75.6%, 95% CI 74.0% to 77.2% for QFracture®. FRAX® maintained a higher accuracy for MOF (71.6%, 95% CI 70.6% to 72.6%) than QFracture® (61.1%, 95% CI 60.0% to 62.2%).

For peer review only

Appendix 4

Figure E1: Study population flow diagram

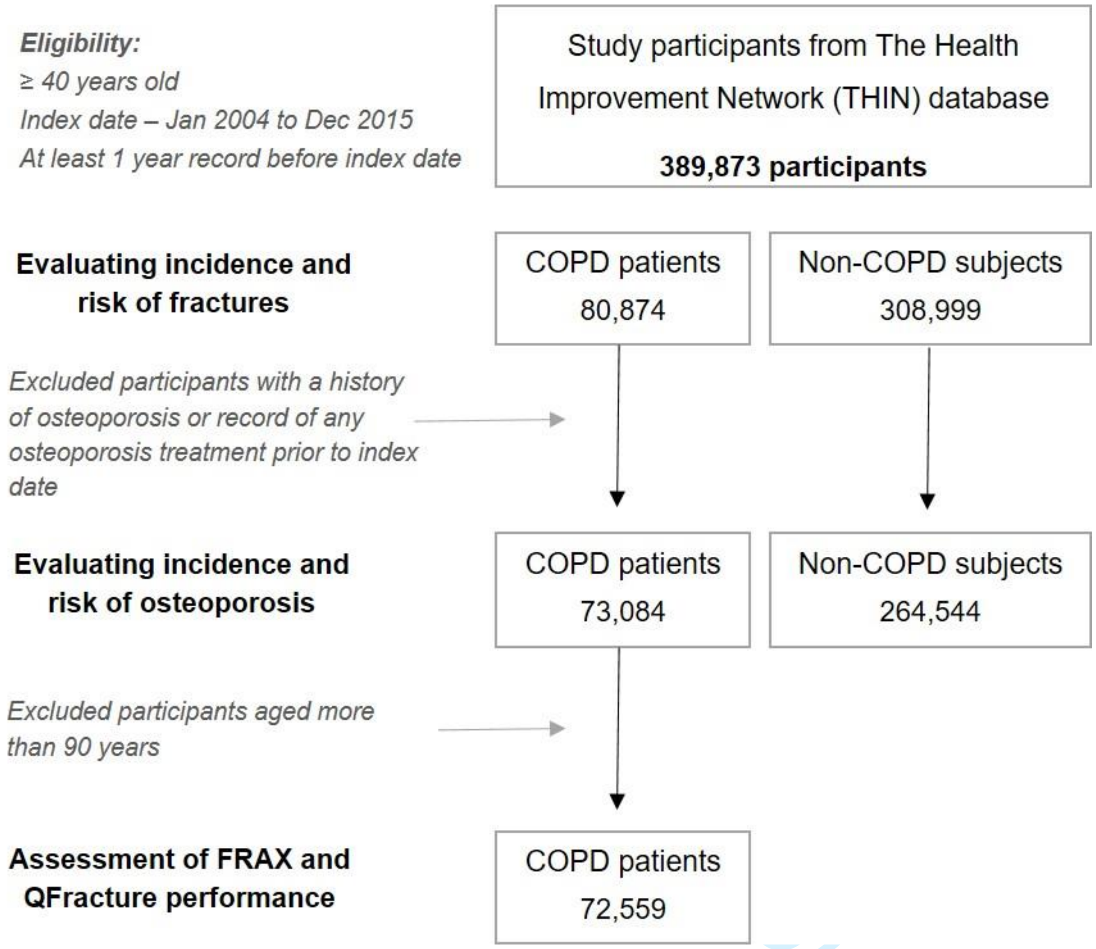

For peer review only - http://bmjopen.bmj.com/site/about/guidelines.xhtml

Appendix 5

Table E1: Risk of osteoporosis in patients with COPD compared with non-COPD subjects

Descriptor	Crude HR (95% CI)	Fully adjusted HR (95% CI)
**COPD**		
Non-COPD subjects	Reference	Reference
COPD patients	1.96 (1.87 – 2.06)	1.13 (1.05 – 1.22)
**Charlson Comorbidity Index**		
Score 0	Reference	Reference
Score 1	1.27 (1.18 – 1.36)	1.14 (1.06 – 1.23)
Score 2	1.34 (1.24 – 1.44)	1.27 (1.17 – 1.37)
Score 3	1.41 (1.28 – 1.55)	1.29 (1.17 – 1.42)
Score 4 & more	1.48 (1.33 – 1.64)	1.44 (1.29 – 1.61)
**Body Mass Index** (kg/m^2)		
Underweight (<18.5)	1.93 (1.64 – 2.27)	1.91 (1.63 – 2.25)
Normal (18.5 – 24.9)	Reference	Reference
Overweight (25 – 29.9)	0.64 (0.60 – 0.69)	0.63 (0.58 – 0.67)
Obese (≥ 30)	0.47 (0.43 – 0.51)	0.45 (0.41 – 0.48)
No record	0.50 (0.46 – 0.53)	0.57 (0.52 – 0.61)
**Smoking status**		
Never	Reference	Reference
Ex	1.01 (0.95 – 1.08)	1.02 (0.95 – 1.09)
Current	1.23 (1.13 – 1.33)	1.15 (1.06 – 1.25)
Unknown	0.69 (0.64 – 0.74)	0.77 (0.71 – 0.83)
**Oral Corticosteroid Use**		
Unexposed	Reference	Reference
Exposed	2.79 (2.56 – 3.05)	1.91 (1.73 – 2.10)
**Inhaled Corticosteroid Use**		
No	Reference	Reference
Yes	1.35 (1.26 – 1.45)	1.24 (1.15 – 1.34)

HR – Hazard ratio; CI – Confidence interval

Crude HR – Cox regression model derived HR adjusted for age, sex, and GP practice

The adjusted Hazard Ratio (aHR) was 1.13, 95% CI 1.05 to 1.22, p<0.0001 – the multivariate Cox regression model derived aHR was adjusted for age, sex, GP practice, Charlson comorbidity index, body mass index, smoking status, inhaled corticosteroid use, and cumulative oral corticosteroid use.

For peer review only

Appendix 6

Table E2: Baseline characteristics of patients with COPD aged 40-90 years with no prior diagnosis of osteoporosis or prescription of any anti-resorptive treatment

Descriptor	COPD patients	
	n = 72,559	%
**Mean age at index date (years, SD)**	66.1 (10.7)	
**Gender**		
Female	31,885	43.9
**MRC Dyspnoea Scale** (1 Year either side of diagnosis)		
1	8,882	12.2
2	17,718	24.4
3	9,257	12.8
4 & 5	4,346	6.0
No record	32,356	44.6
**Charlson Comorbidity Index Score**		
0	0	0
1	38,573	53.2
2	11,953	16.5
3	11,110	15.3
≥ 4	10,923	15.1
**Body Mass Index (BMI) (kg/m²)**		
Underweight (< 18.5)	2,730	3.8
Normal (18.5 – 24.9)	21,791	30.0
Overweight (25 – 29.9)	21,504	29.6
Obese (≥30)	17,627	24.3
No BMI	8,907	12.3
**Smoking status** (1 Year either side of diagnosis)		
Never smoked	7,062	9.7
Ex-smoker	33,810	46.6
Current smoker	29,949	41.3
Unknown	1,738	2.4

For peer review only

STROBE 2007 (v4) Statement—Checklist of items that should be included in reports of *cohort studies*

Section/Topic	Item #	Recommendation	Reported on page #
**Title and abstract**	1	(*a*) Indicate the study's design with a commonly used term in the title or the abstract	1
		(*b*) Provide in the abstract an informative and balanced summary of what was done and what was found	2
**Introduction**			
Background/rationale	2	Explain the scientific background and rationale for the investigation being reported	5
Objectives	3	State specific objectives, including any pre-specified hypotheses	5
**Methods**			
Study design	4	Present key elements of study design early in the paper	6
Setting	5	Describe the setting, locations, and relevant dates, including periods of recruitment, exposure, follow-up, and data collection	6
Participants	6	(*a*) Give the eligibility criteria, and the sources and methods of selection of participants. Describe methods of follow-up	6
		(*b*) For matched studies, give matching criteria and number of exposed and unexposed	6
Variables	7	Clearly define all outcomes, exposures, predictors, potential confounders, and effect modifiers. Give diagnostic criteria, if applicable	6,7
Data sources/ measurement	8*	For each variable of interest, give sources of data and details of methods of assessment (measurement). Describe comparability of assessment methods if there is more than one group	6,7
Bias	9	Describe any efforts to address potential sources of bias	7
Study size	10	Explain how the study size was arrived at	-
Quantitative variables	11	Explain how quantitative variables were handled in the analyses. If applicable, describe which groupings were chosen and why	7
Statistical methods	12	(*a*) Describe all statistical methods, including those used to control for confounding	7
		(*b*) Describe any methods used to examine subgroups and interactions	-
		(*c*) Explain how missing data were addressed	6, Appendix 2
		(*d*) If applicable, explain how loss to follow-up was addressed	-
		(*e*) Describe any sensitivity analyses	7
**Results**			
Participants	13*	(a) Report numbers of individuals at each stage of study—eg numbers potentially eligible, examined for eligibility, confirmed	9

For peer review only

		eligible, included in the study, completing follow-up, and analysed	
		(b) Give reasons for non-participation at each stage	-
		(c) Consider use of a flow diagram	-
Descriptive data	14*	(a) Give characteristics of study participants (eg demographic, clinical, social) and information on exposures and potential confounders	9, Table 1 (17)
		(b) Indicate number of participants with missing data for each variable of interest	Table 1 (19)
		(c) Summarise follow-up time (eg, average and total amount)	9
Outcome data	15*	Report numbers of outcome events or summary measures over time	9, 10
Main results	16	(*a*) Give unadjusted estimates and, if applicable, confounder-adjusted estimates and their precision (eg, 95% confidence interval). Make clear which confounders were adjusted for and why they were included	9, 10, Table 2 (20), Appendix 5
		(*b*) Report category boundaries when continuous variables were categorized	Appendix 2
		(*c*) If relevant, consider translating estimates of relative risk into absolute risk for a meaningful time period	-
Other analyses	17	Report other analyses done—eg analyses of subgroups and interactions, and sensitivity analyses	9,10,11
**Discussion**			
Key results	18	Summarise key results with reference to study objectives	12
**Limitations**			
Interpretation	20	Give a cautious overall interpretation of results considering objectives, limitations, multiplicity of analyses, results from similar studies, and other relevant evidence	12-14
Generalisability	21	Discuss the generalisability (external validity) of the study results	14
**Other information**			
Funding	22	Give the source of funding and the role of the funders for the present study and, if applicable, for the original study on which the present article is based	15

*Give information separately for cases and controls in case-control studies and, if applicable, for exposed and unexposed groups in cohort and cross-sectional studies.

Note: An Explanation and Elaboration article discusses each checklist item and gives methodological background and published examples of transparent reporting. The STROBE checklist is best used in conjunction with this article (freely available on the Web sites of PLoS Medicine at http://www.plosmedicine.org/, Annals of Internal Medicine at http://www.annals.org/, and Epidemiology at http://www.epidem.com/). Information on the STROBE Initiative is available at www.strobe-statement.org.

For peer review only

BMJ Open

Predicting Fracture Risk in Patients with Chronic Obstructive Pulmonary Disease: A UK-based Population-based Cohort Study

Journal:	*BMJ Open*
Manuscript ID	bmjopen-2018-024951.R2
Article Type:	Research
Date Submitted by the Author:	23-Jan-2019
Complete List of Authors:	Akyea, Ralph; University of Nottingham, Nottingham Respiratory Research Unit, NIHR Nottingham Biomedical Research Centre; University of Nottingham, Division of Epidemiology & Public Health<br>McKeever, Tricia; University of Nottingham, Nottingham Respiratory Research Unit, NIHR Nottingham Biomedical Research Centre; University of Nottingham, Division of Epidemiology & Public Health<br>Gibson, Jack; University of Nottingham, Division of Epidemiology & Public Health<br>Scullion, Jane; Institute for Lung Health, University Hospitals of Leicester Glenfield Site<br>Bolton, Charlotte; University of Nottingham, Nottingham Respiratory Research Unit, NIHR Nottingham Biomedical Research Centre
**Primary Subject Heading**:	Respiratory medicine
Secondary Subject Heading:	Epidemiology
Keywords:	Fracture, COPD, fracture risk prediction tool, osteoporosis

SCHOLARONE™
Manuscripts

Predicting Fracture Risk in Patients with Chronic Obstructive Pulmonary Disease: A UK-based Population-based Cohort Study

Ralph K Akyea[1, 2]; Tricia M McKeever[1, 2]; Jack E Gibson[2]; Jane E Scullion[3]; Charlotte E Bolton[1]

Authors affiliations:

[1] Nottingham Respiratory Research Unit, NIHR Nottingham Biomedical Research Centre, School of Medicine, University of Nottingham, UK.

[2] Division of Epidemiology and Public Health, School of Medicine, University of Nottingham, UK.

[3] University Hospitals of Leicester Glenfield Site, Institute for Lung Health, Leicester, UK.

Corresponding author information:

Professor Charlotte E Bolton,

Nottingham Respiratory Research Unit, NIHR Nottingham Biomedical Research Centre, School of Medicine, University of Nottingham, City Hospital Campus, Hucknall Road, Nottingham, NG5 1PB, UK.

Tel: +44 (0)115 8231710

Email: charlotte.bolton@nottingham.ac.uk

Keywords:

Fracture, osteoporosis, COPD, fracture risk prediction tool

Word counts:

Abstract: 297

Main text: 3,024

For peer review only

ABSTRACT

Objectives To assess incidence of hip fracture and all major osteoporotic fractures (MOF) in patients with COPD compared to non-COPD patients and to evaluate the use and performance of fracture risk prediction tools in patients with COPD. To assess the prevalence and incidence of osteoporosis.

Design Population-based cohort study

Setting UK General Practice health records from The Health Improvement Network database

Participants Patients with an incident COPD diagnosis from 2004-2015 and age, sex and general practice matched non-COPD patients were studied.

Outcomes Incidence of fracture (hip alone and all MOF); accuracy of fracture risk prediction tools in COPD; prevalence and incidence of coded osteoporosis.

Methods: Cox proportional hazards models were used to assess the incidence rates of osteoporosis, hip fracture and MOF (hip, proximal humerus, forearm and clinical vertebral fractures). The discriminatory accuracies (area under the receiver operating curve [ROC]) of fracture risk prediction tools (FRAX® and QFracture®) in COPD were assessed.

Results Patients with COPD (n=80,874) were at increased risk of fracture (both hip alone and all MOF) compared to non-COPD patients (n=308,999), but this was largely mediated through oral corticosteroid use, BMI and smoking. Retrospectively calculated ROC for MOF in COPD were FRAX®: 71.4% (95% CI: 70.6 to 72.2%), QFracture®: 61.4% (95% CI: 60.5 to 62.3%) and for hip fracture alone, both 76.1% (95% CI: 74.9 to 77.2%). Prevalence of coded osteoporosis was greater for patients (5.7%) compared to non-COPD patients (3.9%), $p<0.001$. Incidence of osteoporosis was increased in patients with COPD (n=73,084) compared to non-COPD patients (n=264,544), (adjusted hazard ratio, 1.13, 95% CI 1.05 to 1.22).

For peer review only

For peer review only - http://bmjopen.bmj.com/site/about/guidelines.xhtml

Conclusion Patients with COPD are at increased risk of fractures and osteoporosis. Despite this, there is no systematic assessment of fracture risk in clinical practice. Fracture risk tools identify those at high-risk of fracture in patients with COPD.

For peer review only

Strengths and limitations of this study

- This study examined electronic health records from a large, nationally representative sample of the UK population.
- A wide range of potential confounders were evaluated and adjusted for in the analyses.
- For the assessment of the fracture prediction tools, the population of patients with COPD used was large, with many fracture (hip alone and all MOF) events, and it included both men and women.
- Read codes recorded in UK primary care do not capture free text from consultations but capture new diagnoses such as diagnosed osteoporosis and significant fractures such as those classed as MOF.
- The incidence of diagnosed osteoporosis based on clinical codes, may reflect an underestimation of the true risk of osteoporosis since bone mineral density is not systematically assessed.

For peer review only

INTRODUCTION

Osteoporosis in both male and female patients with COPD is firmly established as one of the core comorbid conditions.[1,2] Over the last decade, it has become clear that osteoporosis is not just an end-stage COPD problem[3] nor just in those on maintenance oral corticosteroids (OCS), but it also occurs in a large proportion of those with mild-moderate airflow obstruction and even in steroid naïve patients.[4,5] The Global Initiative for Chronic Obstructive Pulmonary Disease (GOLD) strategy recommends that osteoporosis co-existence should be considered in COPD [1] and the UK National Institute for Health and Care Excellence (NICE) Guidelines on osteoporosis considers COPD as a secondary cause of osteoporosis encouraging use of fracture prediction tools.[6]

The causes for osteoporosis in COPD are likely multiple and cumulative, including age, smoking exposure, inactivity, low body mass index (BMI), systemic inflammation and the frequent use of OCS.[7] The clinical implications of osteoporosis include increased risk of fractures, poor quality of life, pain and further deterioration in lung function.[8,9] Osteoporosis can also remain undiagnosed as asymptomatic for many years.[10] Fractures are a function of trauma sustained, such as falls which are common in COPD [11], and the quality and architecture of bone. Fractures contribute further pain, poor quality of life, increased mortality and confer a substantial economic burden on health systems, patients and their families.[12,13] Given this, the individual risk of a future fracture in patients with COPD is crucial to determine in patient care and to treat accordingly.

Fracture risk prediction tools based on clinical and personal characteristics have been developed over the years to guide investigation and management of those identified to be at high risk of osteoporotic fractures, worldwide. These include for the UK (and many other regions), FRAX® and QFracture®.[6]

The full extent of fracture risk assessment in patients with COPD is not fully established. The aim of this study was to assess incidence of hip fracture alone or all major osteoporotic fractures (MOF) in patients with COPD compared to non-COPD patients and to evaluate the use and performance of fracture risk prediction tools in patients. Further, to assess the prevalence of coded osteoporosis up to the time of COPD diagnosis and the incidence of osteoporosis.

For peer review only

METHODS

Information for this cohort study was obtained from The Health Improvement Network (THIN), an anonymised primary care database representing 6.2% of the total UK population.[14]

The study population consisted of patients 40 years and over with a new Read coded COPD diagnosis during the data collection period 1/1/04-31/12/15, with at least 1 year of record prior to COPD diagnosis.[15] Each patient was matched by age, sex and GP practice to up to four patients without a history of COPD to generate a matched cohort and assigned the same index date.

Follow up was from the index date to the first record of either the occurrence of the outcome of interest (fracture/osteoporosis), the date of transfer of the patient out of the practice area, death or end of THIN data collection. Diagnoses for osteoporosis – classed as coded osteoporosis *(Appendix 1)*, hip fracture alone and all MOF (comprising fracture of the hip, proximal humerus, forearm or clinically symptomatic vertebra/spine), coded using the standard Read code classification were used.[16]

A series of explanatory variables [6,17] determined at baseline (prior to or at index date) included: Charlson Comorbidity Index (CCI) score,[18] Townsend social deprivation score, recorded history of fall, prior fractures, parental history of fall/osteoporosis, relevant comorbidities and secondary causes of osteoporosis as defined in the FRAX® questionnaire.[19] Records for smoking status, alcohol use, MRC Dyspnoea scale, BMI, and use of specific prescription drugs were restricted to a defined time period.

To account for use of OCS, individual follow-up time was divided into periods during which participants were considered exposed, or not exposed, to OCS (a binary variable). Exposed periods started from prescription date until the first gap of more than 90 days between prescriptions; with individuals considered unexposed from the 91st day onwards. Individuals were considered exposed at study entry if they had received a relevant prescription within 90 days prior. The effect of exposure was assumed to be constant, and not cumulative, over time.

For peer review only - http://bmjopen.bmj.com/site/about/guidelines.xhtml

Input variables included clinical status, prescription drug use, and demographic characteristics, according to the variables/definitions used in both FRAX® and QFracture® tools,[19,20], additional detail on the method is provided in an online data supplement *(Appendix 2)*. Imputation was used for missing variables.

The 10-year risk score for hip fracture alone and all MOF according to QFracture® (version 2017.0.0.0) and FRAX® for UK (without bone mineral density (BMD)information) (desktop version 3.12) were calculated for patients with COPD, aged 40-90 years old. A complete case sensitivity analysis without imputed variables was also performed (Appendix 3).

Statistical analyses

Incidence rates were calculated for both groups using Cox proportional hazards regression to estimate hazard ratios (HRs) of coded osteoporosis and fracture (hip alone and all MOF) risks. Conditional analysis to account for matching by age, sex and GP practice was done. Confounders were included in the final fully adjusted multivariable models when independently changing the HRs for osteoporosis/fracture by at least 5%. A former osteoporosis diagnosis or antiresorptive treatment prior to COPD diagnosis excluded that subject from analyses related to either osteoporosis incidence or risk *(Appendix 4)*. In addition to evaluating incidence in the whole cohort, separate sub-analyses excluded a) patients with COPD and no documented smoking history together with their matched non-COPD patients and b) those with no prior record of osteoporosis.

To evaluate FRAX® and QFracture®, the outcome was treated as a binary variable (fracture or no fracture). Fracture risk probabilities were categorised based on recommended treatment thresholds (≥ 20% for MOF and ≥3% for hip fracture).[21] To evaluate the overall ability of each tool to discriminate (performance) between those at low and high risks, the area under the receiver operating characteristic (ROC) curve was calculated. Sensitivity, specificity, positive and negative predictive values were calculated. Survival analysis was performed and Kaplan-Meier plots comparing the MOF incidence were generated.

All statistical analyses were performed using Stata 15.0 (StataCorp LP).

For peer review only

Patient involvement

The results and implications of previous research from the team on systematic assessment of osteoporosis in patients with COPD [4] has been discussed extensively in previous patient meetings. Whilst this and other literature has strengthened the GOLD strategy recommendations,[1] evaluation of clinical services would suggest systematic assessment is not done in patients. More recently, patients with COPD out-patient clinics have approached the principal investigator at the time of their "ad hoc osteoporosis" diagnosis to ask why this was not investigated at or closer to COPD diagnosis and how osteoporosis could be assessed. This has led to the development of this grant application with significant patient input in the design and context. The results have been discussed back with representatives on the respiratory research panel. Given the implications for clinical practice, the findings have been discussed extensively at the PPI meeting and a Breathe Easy meeting in early 2018. A lay summary has been developed for the patient newsletter (n>700) and website. In the meantime, members of the respiratory research panel are assisting the PI in planning future work regarding implementation.

For peer review only

RESULTS

The baseline characteristics are shown in Table 1. A total of 80,874 eligible patients with COPD and 308,999 matched non-COPD patients were identified. The median follow-up period was 5 years for both patients with COPD and non-COPD patients.

Osteoporosis at index date and incidence

Prevalence of coded osteoporosis up to the index date was greater for patients with COPD (5.7%) compared to non-COPD patients (3.9%), $p<0.001$. Within 1 year (before and after) of the index date, 1,504 (1.86%) patients with COPD had a new recorded diagnosis of osteoporosis compared to 3,059 (1.12%) in matched non-COPD patients, p <0.001. 3,186 (3.94%) of patients with COPD had a diagnosis of osteoporosis more than a year prior to index date compared to 8,822 (2.86%) for the matched controls p <0.001.

1,457 (1.80%) patients with COPD compared to 3,694 (1.20%) non-COPD patients, had a record of any diagnostic assessment for osteoporosis, recorded within 1 year (before and after) of the index date, ($p<0.001$).

Demographics remained similar after excluding those with former coded osteoporosis. Patients with COPD (n=73,084) compared to non-COPD patients (n=264,544) were significantly more likely to have incident diagnosis of osteoporosis (hazard ratio (HR), 1.96; 95% confidence interval [CI] 1.87 to 2.05; *Appendix 5*).

Incidence of Fracture

There was a significantly increased risk of MOF, hazard ratio of 1.60 (95% CI 1.52 to 1.69) and hip fractures alone: 1.67 (95% CI 1.56 to 1.80) in patients with COPD compared to non-COPD patients. In the fully adjusted models the associations were diminished (Table 2). Smoking status altered the effect between COPD and fracture the most, followed by BMI, CCI score and OCS.

Sensitivity analysis with participants with no former osteoporosis showed similar results. The risk of MOF was also similar when evaluated in only patients with COPD with a documented prior history of smoking and their matched controls. However, here, the risk of hip fracture remained significantly increased in the adjusted model compared to non-COPD patients (aHR, 1.13; 95% CI 1.004 to 1.280; p-value: 0.043).

For peer review only - http://bmjopen.bmj.com/site/about/guidelines.xhtml

Fracture risk prediction tools in COPD

Only 1074 (1.33%) of patients with COPD had a FRAX® assessment READ coded ever documented in the records and 12 patients had a READ coded QFracture® assessment. Within 1 year (before and after) of index date, 248 (0.31%) of patients with COPD had a FRAX® and only 1 patient a QFracture®.

The final population for the discriminatory accuracy analysis comprised 72,559 patients aged 40-90 years with COPD and no prior diagnosis of osteoporosis or prescription of any anti-resorptive treatment (*demographics in Appendix 6*). This included 4,605 (6.4%) patients who experienced any MOF and 1,444 (2.0%) who experienced a hip fracture.

When the FRAX® and QFracture® scores were calculated for patients with COPD, for hip fracture there were 29,035 (40.0%) patients who had a risk ≥3% using FRAX® and 33,065 (45.6%) patients using the QFracture®. For any MOF, 6,221 (8.6%) of patients had a risk ≥20% using FRAX® and 9,546 (13.2%) patients using QFracture®.

Both risk tools had a similar discriminatory accuracy for hip fracture (FRAX® 76.1%, 95% CI 74.9 to 77.2% and QFracture® 76.1%, 95% CI 74.9 to 77.2%). FRAX®, however, had a higher accuracy for MOF (71.4% 95% CI 70.6% to 72.2%) than QFracture® (61.4% 95% CI 60.5% to 62.3%). The discriminatory accuracies were better in women than men. The performance of the prediction tools was similar in the patients aged 50-90 years compared to 40-90-year olds.

The sensitivity of the risk scores for any MOF (using >20% risk as cut-off) were similar: FRAX®:25.4% and QFracture®: 25.2%. The sensitivity of the risk scores for hip fracture (using > 3% cut-off) were slightly worse for FRAX®:78.1% compared to 82.1% for QFracture®. The specificity and positive predictive value were better for FRAX® than QFracture®, Table 3.

The association of an increased fracture risk (either FRAX® or QFracture®) with incidence of any MOF is shown in Figure 1.

For peer review only - http://bmjopen.bmj.com/site/about/guidelines.xhtml

DISCUSSION

Using UK primary care electronic health records, we have reported on the burden of fractures in patients with COPD with both hip fracture alone or any MOF increased in patients with COPD compared to age, sex and GP surgery matched patients. Despite the increased fracture risk and recommendations in the NICE osteoporosis guidelines, fracture risk prediction tools are rarely coded. However, where the risk score was retrospectively calculated, the risk prediction tools identify those at risk of hip fracture or any MOF. Therefore, fracture risk prediction and subsequent targeted therapy and management could transform multi-morbidity management of COPD. In addition, we report that the prevalence and incidence of osteoporosis, a risk for fracture, in patients with COPD, is far greater than in non-COPD patients.

Prevalence of osteoporosis varies widely in the different research studies of patients with COPD. This is likely due to the severity of COPD,[4,5] whether osteoporosis was systematically sought or self-reported [4,22], and whether patients included were on OCS.[3] A prevalence of 23-32% has been reported where BMD was systematically performed in COPD [23][4], while 14% of patients with COPD self-reported osteoporosis compared to 5% in those without COPD.[22] The prevalence of coded osteoporosis in the GP health records presented here was, however, far lower at 5.7% than the reported prevalence from clinical studies when osteoporosis and BMD are systematically assessed. This raises the question of subclinical, undiagnosed osteoporosis disease leading to a missed opportunity for intervention and strengthening the need for a systematic assessment especially when cost-efficient anti-resorptive treatment is available.[24]

There is growing consensus on COPD being a secondary cause of osteoporosis, including within the NICE clinical guideline on osteoporosis where fracture risk prediction tools are recommended, yet in practice seem rarely done.[6] Whilst osteoporosis in itself leads to pain and poor quality of life,[25] ultimately osteoporosis treatment aims to reduce the risk of fracture.[24,26] Risk factors for fracture include osteoporosis but also falls, which, are greater in patients with COPD.[11,27] Whilst the increased risk of fractures in COPD has previously been considered,[28] they have not assessed incidence from time of COPD diagnosis or only reported as part of a larger study of post-menopausal women [29] or analysed the history of obstructive airway disease (both COPD and asthma together) before

For peer review only

the index date of osteoporotic fracture in both cases and controls over the age of 18 years.[30]

Little is known about the use of fracture risk assessment tools in patients with COPD. A number of validation studies have performed independent assessments to predict subsequent fracture in the general population.[31,32] The studies differ widely in sample size, methodology, and techniques used to assess performance.[33] Discrimination for FRAX® (without BMD incorporation) and QFracture® have both been reported as good.[31,34,35] The results from this COPD study are comparable to the general population validation studies,[31,34,35] however, the AUC for MOF using QFracture® was lower than that reported in other studies. Similar to findings from studies based on general population, the discrimination from our study was better in women than men and better for hip fracture than MOF.[36] The discrimination appeared similar within the 50-90 year-old group when compared to the 40-90 year-olds. Despite the two tools having differences in their approach to calculating fracture risks, both predict fractures satisfactorily in patients with COPD. Despite the sensitivity and positive predictive values being far from ideal, sensitivity reported in our study are comparable to those published in studies using a general population.[31,35] Although a bespoke COPD tool could be adapted in the future, the use of one of the established fracture risk scores in the meantime provides the opportunity to systematically identify and intervene. Such tools are incorporated into primary care medical record systems and utilised in a number of other disease areas. Available fracture prevention therapy (anti-resorptive agents) are very effective, safely yielding 40-60% reduction in the risk of fracture.[26] These medications are cost-effective in high-risk patients –reduces morbidity, mortality and health care cost associated with osteoporotic fractures.[24] The fracture prediction tools could be integrated into COPD annual assessments or at COPD diagnosis. Identification of patients at high risk is valuable information to guide and optimise treatment options. Though the optimal pathways for this integration is required.

The use of OCS has been considered to be a major contributory factor in the development of osteoporosis. However, osteoporosis has been reported in patients with no OCS use.[4,5] Other known osteoporosis risk factors are also likely to contribute in patients with COPD including smoking, a low BMI, physical inactivity and systemic inflammation. Some of these risk factors could be moderated through education, smoking cessation, pulmonary rehabilitation and lifestyle changes.[37,38] Recognition of the scale and impact of fracture risk draws further necessary attention

For peer review only - http://bmjopen.bmj.com/site/about/guidelines.xhtml

to these interventions to aim to prevent and reduce risks, alongside appropriate pharmacotherapy.

The study had several strengths in its methods, analyses, findings, and implications for clinical practice. Firstly, this research was population-based and compared patients with COPD with age-sex matched control patients from the same general practice. Its external validity and hence generalisability was high because THIN database is representative of the UK population.[14] There was a substantial duration of follow-up. A wide range of potential confounders were also evaluated and adjusted for in the analyses.

For the assessment of the fracture prediction tools, the population of patients with COPD used was large, with many fracture events, and included both men and women. This minimised the likelihood of a selection bias. The assessments of the prediction tools were done using the same population, therefore minimising the effect of confounding for a difference in performance. We are presently not aware of studies that have determined the performance of the recommended fracture prediction tools in the sub-population of patients with COPD. The dataset was using UK electronic health records but is likely representative of other countries in representing the scale of the problem and the utility of the risk prediction scores.

Regarding limitations, some variables might be subject to information or reporting bias as Read codes recorded in databases do not capture free text from consultations. Such variables include patient reported alcohol intake, use of cigarettes or their awareness of relevant family history. The possibility of residual confounding can also not be excluded as risk factors such as physical activity, diet and ethnicity could not be adjusted for in the analyses. An accepted definition of fractures types was used; however, it is difficult to determine the cause of fracture based simply on fracture site, with no additional information. Unlike studies which assess BMD systematically, this is not currently done in clinical practice, nor are the fracture risk scores routinely calculated as highlighted here. Therefore, the incidence of osteoporosis based on clinical codes likely reflects an underestimation of the true increased incidence/risk of osteoporosis.

In summary, despite validated fracture risk prediction tools, there was very little assessment of the increased fracture risk in patients with COPD. However, on retrospective calculation of fracture risk, the tools identify those patients with COPD

For peer review only

at greatest risk of fracture. Identification with a systematic assessment of bone health and addressing prevention and treatment of those at greatest risk of fracture has the potential to improve outcomes for patients with COPD.

For peer review only

For peer review only - http://bmjopen.bmj.com/site/about/guidelines.xhtml

Acknowledgements

With grateful thanks to Prof J Hippisley-Cox and Mr S Hippisley-Cox for use of the QFracture®.

Competing interests

All authors have completed the Unified Competing Interest form (available on request from the corresponding author) and declare: CEB, TMM, JES received an investigator sponsored study grant from Pfizer for the submitted work; CEB reports grants from MRC/Association of British Pharmaceutical Industry (ABPI), TSB, GSK and other support from Chiesi and Boehringer, outside the submitted work; JES reports personal fees from Astra Zeneca, Boehringer-Ingelheim, Nutricia, Chiesi, Sandoz, Novartis, Pfizer, MIMS, RCGP, Cogora and other support from PCRS-UK, Education for Health, Teva and NICE outside the submitted work; no financial relationships with any organisations that might have an interest in the submitted work in the previous three years, no other relationships or activities that could appear to have influenced the submitted work.

Author contributions

CEB, TMM and JES designed study concept and design and are grant holders. RKA conducted the main statistical analysis and wrote the first draft of the manuscript. JEG prepared the THIN data extracts used and assisted with analysis. All authors contributed to the interpretation of the data, writing of the manuscript and critical revisions.

CEB is guarantor.

Ethical approval

The study was approved by an independent Scientific Review Committee (SRC), 16THIN029.

Funding

This study was funded by a COPD "Open Air" research grant from Pfizer.

The funders had no role in study design, data collection and analysis, decision to publish or preparation of the manuscript. CEB and TMM are supported by the NIHR Nottingham BRC. The views expressed are those of the authors and not necessarily those of the NHS, the NIHR or the Department of Health.

Data sharing

No additional data available.

For peer review only

REFERENCES

1 Global Strategy for the Diagnosis Management and Prevention of COPD. Global Initiative for Chronic Obstructive Lung Disease (GOLD) 2017. http://goldcopd.org/gold-2017-global-strategy-diagnosis-management-prevention-copd/ (accessed 13 Apr 2017).

2 Barnes PJ, Celli BR. Systemic manifestations and comorbidities of COPD. *Eur Respir J* 2009;**33**:1165–85. doi:10.1183/09031936.00128008

3 Shane E, Silverberg SJ, Donovan D, *et al.* Osteoporosis in lung transplantation candidates with end-stage pulmonary disease. *Am J Med* 1996;**101**:262–9.

4 Bolton CE, Ionescu AA, Shiels KM, *et al.* Associated Loss of Fat-free Mass and Bone Mineral Density in Chronic Obstructive Pulmonary Disease. *Am J Respir Crit Care Med* 2004;**170**:1286–93. doi:10.1164/rccm.200406-754OC

5 Duckers JM, Evans BA, Fraser WD, *et al.* Low bone mineral density in men with chronic obstructive pulmonary disease. *Respir Res* 2011;**12**:101. doi:10.1186/1465-9921-12-101

6 National Institute for Health and Care Excellence. Osteoporosis: assessing the risk of fragility fracture. 2012.

7 Lehouck A, Boonen S, Decramer M, *et al.* COPD, Bone Metabolism, and Osteoporosis. *Chest* 2011;**139**:648–57. doi:10.1378/chest.10-1427

8 Gjertsen J-E, Baste V, Fevang JM, *et al.* Quality of life following hip fractures: results from the Norwegian hip fracture register. *BMC Musculoskelet Disord* 2016;**17**. doi:10.1186/s12891-016-1111-y

9 Jameson JL, De Groot LJ. *Endocrinology: Adult and Pediatric E-Book*. Elsevier Health Sciences 2015.

10 Peppas NA, Hilt JZ, Thomas JB. *Nanotechnology in Therapeutics: Current Technology and Applications*. Horizon Bioscience 2007.

11 Hakamy A, Bolton CE, Gibson JE, *et al.* Risk of fall in patients with COPD. *Thorax* 2018;**73**:1079–80. doi:10.1136/thoraxjnl-2017-211008

12 Coughlan T, Dockery F. Osteoporosis and fracture risk in older people. *Clin Med* 2014;**14**:187–91. doi:10.7861/clinmedicine.14-2-187

13 Roux C, Wyman A, Hooven FH, *et al.* Burden of non-hip, non-vertebral fractures on quality of life in postmenopausal women. *Osteoporos Int* 2012;**23**:2863–71. doi:10.1007/s00198-012-1935-8

14 Blak BT, Thompson M, Dattani H, *et al.* Generalisability of The Health Improvement Network (THIN) database: demographics, chronic disease prevalence and mortality rates. *Inform Prim Care* 2011;**19**:251–5.

15 Quint JK, Müllerova H, DiSantostefano RL, *et al.* Validation of chronic obstructive pulmonary disease recording in the Clinical Practice Research Datalink (CPRD-GOLD). *BMJ Open* 2014;**4**:e005540. doi:10.1136/bmjopen-2014-005540

16 National Health Service. Read Codes. 2017.https://digital.nhs.uk/services/terminology-and-classifications/read-codes (accessed 29 Dec 2018).

17 Scottish Intercollegiate Guidelines Network (SIGN). Management of osteoporosis and the prevention of fragility fractures. Edinburgh: 2015.

18 Lix LM, Quail J, Teare G, *et al.* Performance of comorbidity measures for predicting

For peer review only

For peer review only - http://bmjopen.bmj.com/site/about/guidelines.xhtml

outcomes in population-based osteoporosis cohorts. *Osteoporos Int* 2011;**22**:2633–43. doi:10.1007/s00198-010-1516-7

19 World Health Organization Collaborating Center for Metabolic Bone Diseases. FRAX® WHO Fracture Risk Assessment Tool. 2011.https://www.sheffield.ac.uk/FRAX/tool.jsp (accessed 18 Sep 2017).

20 ClinRisk Ltd. QFracture-2016® risk calculator. http://www.qfracture.org/ (accessed 20 Sep 2017).

21 Unnanuntana A, Gladnick BP, Donnelly E, *et al.* The Assessment of Fracture Risk. *J Bone Jt Surgery-American Vol* 2010;**92**:743–53. doi:10.2106/JBJS.I.00919

22 Miller J, Edwards LD, Agustí A, *et al.* Comorbidity, systemic inflammation and outcomes in the ECLIPSE cohort. *Respir Med* 2013;**107**:1376–84. doi:10.1016/j.rmed.2013.05.001

23 Ferguson GT, Calverley PMA, Anderson JA, *et al.* Prevalence and progression of osteoporosis in patients with COPD: results from the TOwards a Revolution in COPD Health study. *Chest* 2009;**136**:1456–65.

24 National Institute for Health and Care Excellence. Bisphosphonates for treating osteoporosis. 2017.

25 Kanis JA, Johnell O, Oden A, *et al.* The risk and burden of vertebral fractures in Sweden. *Osteoporos Int* 2004;**15**:20–6. doi:10.1007/s00198-003-1463-7

26 Papaioannou A, Morin S, Cheung AM, *et al.* 2010 clinical practice guidelines for the diagnosis and management of osteoporosis in Canada: Summary. CMAJ. 2010;**182**:1864–73. doi:10.1503/cmaj.100771

27 Sibley KM, Voth J, Munce SE, *et al.* Chronic disease and falls in community-dwelling Canadians over 65 years old: a population-based study exploring associations with number and pattern of chronic conditions. *BMC Geriatr* 2014;**14**:22. doi:10.1186/1471-2318-14-22

28 Gonzalez A V., Coulombe J, Ernst P, *et al.* Long-term Use of Inhaled Corticosteroids in COPD and the Risk of Fracture. *Chest* 2018;**153**:321–8. doi:10.1016/J.CHEST.2017.07.002

29 Dennison EM, Compston JE, Flahive J, *et al.* Effect of co-morbidities on fracture risk: findings from the Global Longitudinal Study of Osteoporosis in Women (GLOW). *Bone* 2012;**50**:1288–93. doi:10.1016/j.bone.2012.02.639

30 de Vries F, van Staa TP, Bracke MSGM, *et al.* Severity of obstructive airway disease and risk of osteoporotic fracture. *Eur Respir J* 2005;**25**:879–84. doi:10.1183/09031936.05.00058204

31 Dagan N, Cohen-Stavi C, Leventer-Roberts M, *et al.* External validation and comparison of three prediction tools for risk of osteoporotic fractures using data from population based electronic health records: retrospective cohort study. *BMJ* 2017;**356**:i6755. doi:10.1136/BMJ.I6755

32 Tamaki J, Iki M, Kadowaki E, *et al.* Fracture risk prediction using FRAX®: A 10-year follow-up survey of the Japanese Population-Based Osteoporosis (JPOS) Cohort Study. Osteoporos. Int. 2011;**22**:3037–45. doi:10.1007/s00198-011-1537-x

33 Steyerberg EW. *Clinical Prediction Models: A Practical Approach to Development, Validation, and Updating*. Springer 2008. doi:10.1080/10543400903244270

34 Kanis JA, Oden A, Johnell O, *et al.* The use of clinical risk factors enhances the performance of BMD in the prediction of hip and osteoporotic fractures in men and

For peer review only

women. *Osteoporos Int* 2007;**18**:1033–46. doi:10.1007/s00198-007-0343-y

35 Hippisley-Cox J, Coupland C. Derivation and validation of updated QFracture algorithm to predict risk of osteoporotic fracture in primary care in the United Kingdom: prospective open cohort study. *BMJ* 2012;**344**:e3427. doi:10.1136/bmj.e3427

36 Cummings SR, Melton LJ. Epidemiology and outcomes of osteoporotic fractures. *Lancet* 2002;**359**:1761–7. doi:10.1016/S0140-6736(02)08657-9

37 National Institute for Health and Care Excellence, NICE. Chronic obstructive pulmonary disease in over 16s: diagnosis and management. NICE 2010.

38 British Thoracic Society, Pulmonary Rehabilitation Guideline, Group. BTS Guideline on Pulmonary Rehabilitation in Adults. *Thorax* 2013;**68**.

For peer review only

For peer review only - http://bmjopen.bmj.com/site/about/guidelines.xhtml

Table 1: Baseline characteristics of patients with COPD and non-COPD patients

Descriptor	COPD patients		Non-COPD patients		
	n = 80,874	%	*n* = 308,999	%	*p*-value
**Mean age at index date (years, SD)**	66.9 (11.0)		66.5 (10.9)		
**Sex**					0.002
Male	42,799	52.9	161,648	52.3	
Female	38,075	47.1	147,351	47.7	
**Follow-up (years, median, IQR)**	5.28	2.6-8.3	5.24	2.6-8.3	
**MRC Dyspnoea Scale** (1 Year either side of diagnosis)					<0.001
1	9,499	11.8	1,168	0.4	
2	19,466	24.1	1,092	0.4	
3	10,488	13.0	446	0.1	
4 & 5	5,237	6.5	177	0.1	
No record	36,184	44.7	306,116	99.1	
**Charlson Comorbidity Index Score**					<0.001
0	0	0.0	172,566	55.9	
1	41,777	51.7	50,955	16.5	
2	13,506	16.7	42,667	13.8	
3	12,694	15.7	23,546	7.6	
≥ 4	12,897	16.0	19,265	6.2	
**Body Mass Index (BMI) (kg/m²)**					<0.001
Underweight (< 18.5)	3,414	4.2	2,699	0.9	
Normal (18.5 – 24.9)	24,734	30.6	54,267	17.6	
Overweight (25 – 29.9)	23,497	29.1	77,129	25.0	
Obese (≥30)	19,083	23.6	60,280	19.5	
No BMI	10,146	12.6	114,624	37.1	
**Smoking status** (1 Year either side of diagnosis)					<0.001
Never smoked	7,925	9.8	94,800	30.7	
Ex-smoker	38,590	47.7	72,989	23.6	
Current smoker	32,436	40.1	34,691	11.2	
Unknown	1,923	2.4	106,519	34.5	
**History of Falls** (prior to or at diagnosis)					
Personal history	8,969	11.1	26,203	8.5	<0.001
Parental history of fall/osteoporosis	96	0.1	298	0.1	0.076
**Medications** (1 Year either side of diagnosis)					
Oral corticosteroid Use (OCS)	33,618	41.6	19,479	6.3	<0.001
Inhaled Corticosteroid Use	47,574	58.8	21,312	6.9	<0.001

For peer review only

Table 2: Risk of all major osteoporotic fractures (MOF) and hip fractures alone in patients with COPD compared with non-COPD patients

	Number of fractures	Rate/1,000 person-years	HR (95% CI)	Fully adjusted HR (95% CI)
**Major osteoporotic fractures (MOF)**				
Non-COPD patients	6,032	4.32 (4.22 – 4.44)	Reference	Reference
Patients with COPD	2,234	6.64 (6.37 – 6.92)	1.60 (1.52 – 1.69)	1.04 (0.96 – 1.12) [a]
**Hip fracture**				
Non-COPD patients	3,170	2.26 (2.18 – 2.34)	Reference	Reference
Patients with COPD	1,213	3.57 (3.38 – 3.78)	1.67 (1.56 – 1.80)	1.09 (0.98 – 1.21) [b]

HR – Hazard ratio; CI – Confidence interval

HR – conditional regression used to account for matching by age, sex and GP practice.

Fully adjusted:

[a] Multivariable Cox regression model derived HR was adjusted for age, sex, GP practice, Charlson Comorbidity Index, Body Mass Index, smoking status, inhaled corticosteroid use, antidepressant use and cumulative oral corticosteroid use.

[b] Multivariable Cox regression model derived HR was adjusted for age, sex, GP practice, Charlson Comorbidity Index, Body Mass Index, smoking status, inhaled corticosteroid use and cumulative oral corticosteroid use.

For peer review only - http://bmjopen.bmj.com/site/about/guidelines.xhtml

Table 3: Discrimination measures for FRAX® and QFracture® at recommended treatment cut offs for both major osteoporotic fractures (MOF) and hip fractures alone

Discriminatory measures	FRAX®	QFracture®
	**Measure for ≥ 20% risk** (95% CI)	**Measure for ≥ 20% risk** (95% CI)
**All major Osteoporotic fractures (MOF)**		
Sensitivity	25.4% (22.7-28.1%)	25.2% (22.5-27.9%)
Specificity	92.6% (91.0-94.2%)	87.7% (85.7-89.7%)
Positive Predictive Value	18.8% (16.4-21.1%)	12.2% (10.2-14.2%)
Negative Predictive Value	94.8% (93.4-96.2%)	94.5% (93.1-95.9%)
	**Measure for ≥ 3% risk**	**Measure for ≥ 3% risk**
**Hip fracture**		
Sensitivity	78.1% (75.6-80.7%)	82.1% (79.7-84.5%)
Specificity	60.8% (57.8-63.8%)	55.2% (52.1-58.3%)
Positive Predictive Value	3.9% (2.7-5.1%)	3.6% (2.5-4.8%)
Negative Predictive Value	99.3% (98.8-99.8%)	99.3% (98.8-99.8%)

CI – Confidence interval

For peer review only

Figure 1: Kaplan-Meier plots comparing incidence of major osteoporotic fractures (MOF) at various predicted fracture risk categories in patients with COPD using (a) FRAX® and (b) QFracture®

For peer review only

(a)

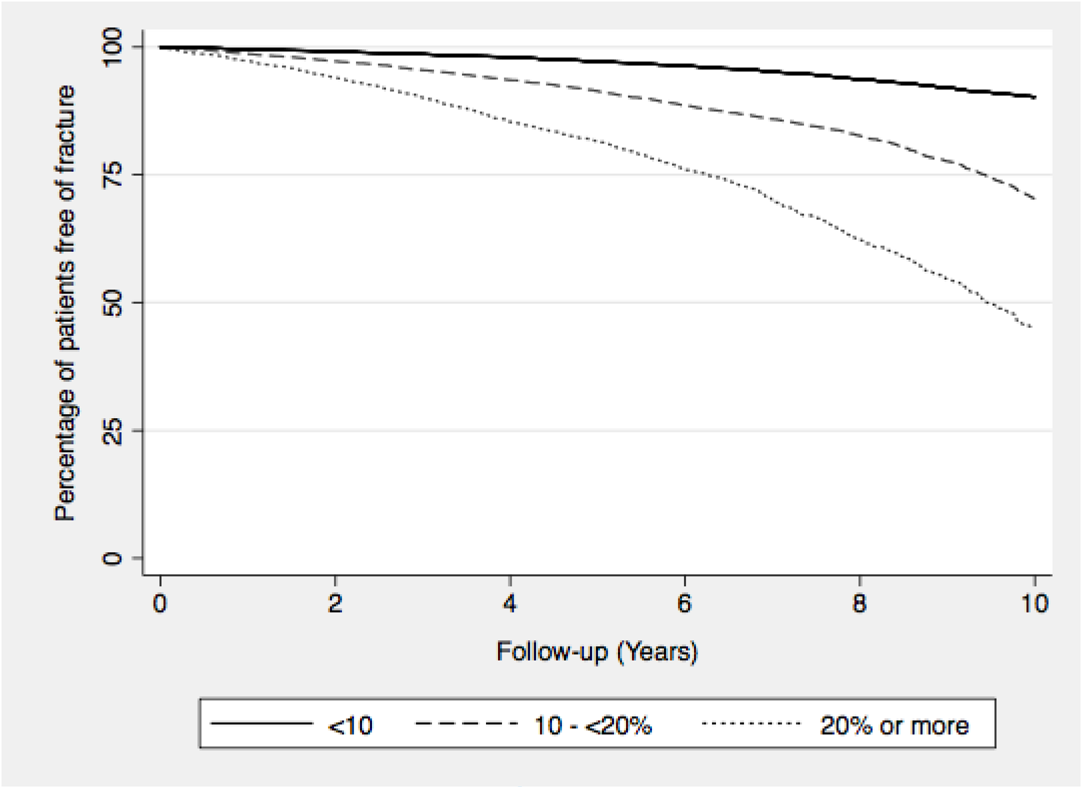
Percentage of patients free of fracture
100
75
50
25
0
0
2
4
6
8
10
Follow-up (Years)
<10
10 - <20%
20% or more

(b)

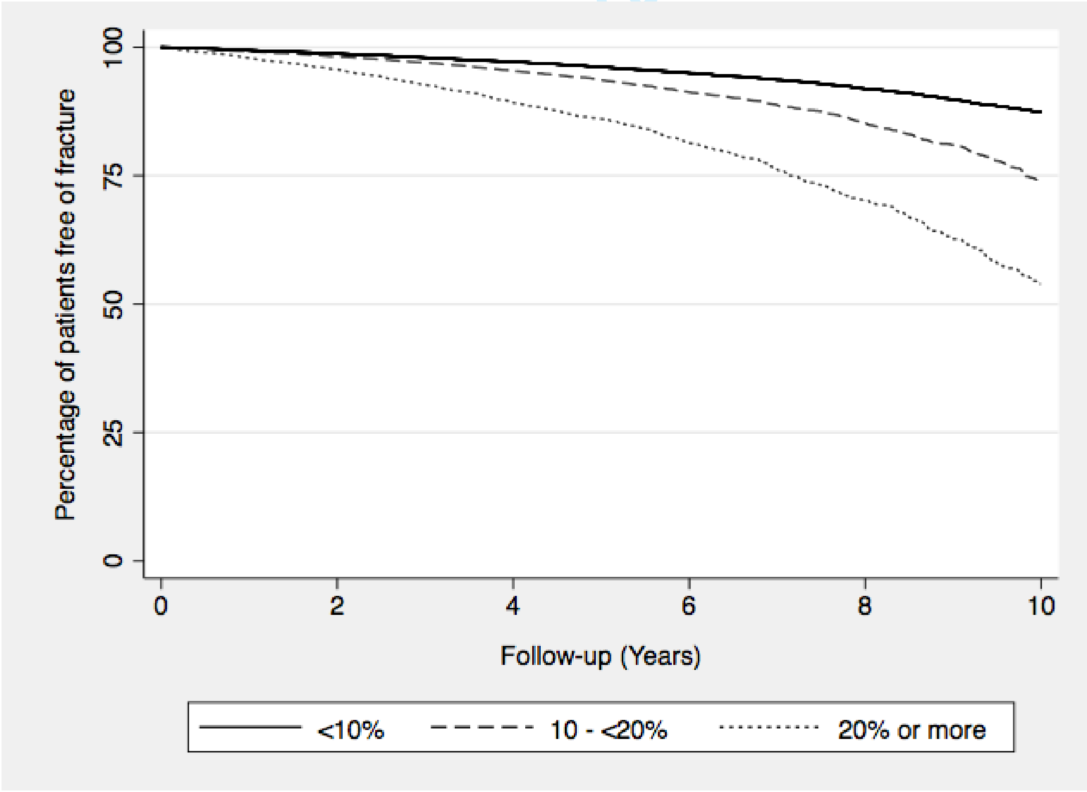
Percentage of patients free of fracture
100
75
50
25
0
0
2
4
6
8
10
Follow-up (Years)
<10%
10 - <20%
20% or more

Appendix 1

Read code definitions for selected input variables

Variable	Read codes
COPD	H3...00, H3...11, H31..00, H310.00, H310000, H310z00, H311.00, H311000, H311100, H311z00, H312.00, H312000, H312011, H312100, H312300, H312z00, H313.00, H31y.00, H31y100, H31yz00, H31z.00, H32..00, H320.00, H320000, H320100, H320200, H320300, H320311, H320z00, H321.00, H322.00, H32y.00, H32y000, H32y100, H32y111, H32y200, H32yz00, H32z.00, H36..00, H37..00, H38..00, H39..00, H3A..00, H3y..00, H3y..11, H3z..00, H3z..11
Osteoporosis	585O.00, 58E4.00, 58E8.00, 58EA.00, 58EE.00, 58EG.00, 58EK.00, 58EM.00, 58ES.00, 58EV.00, 7230A, 7230B, 7230D, 7230PM, 7230PT, N330.00, N330000, N330100, N330200, N330300, N330400, N330500, N330600, N330700, N330800, N330900, N330A00, N330B00, N330C00, N330D00, N330z00, N331200, N331300, N331400, N331500, N331600, N331800, N331900, N331A00, N331B00, N331M00, N331N00, NyuB000, NyuB100, NyuB200, NyuB800
Antiresorptive treatment *(drug code)*	97138998, 99158998, 99158997, 97139998, 96920998, 96789998, 93478998, 97140998, 97218998, 93975992, 83457998, 97064992, 83456998, 96897998, 96020992, 96901998, 95879992, 98249990, 97031992, 98581990, 99018990, 98198990, 62945979, 96737998, 97066992, 97051992, 97780990, 98199990, 61594979, 99261990, 96604992, 92004979, 97248990, 99263990, 94089992, 93127992, 94756992, 91526998, 89828998, 88144998, 88144997, 88225998, 89434998, 93502998, 99862998, 95304998, 93228997, 96904998, 93228998, 95304996, 99862997, 95304997, 93228996, 99864998, 91997998, 91998998, 87933998, 81073998, 61612979, 87155998, 87154998, 88542998, 91378998, 82066998,

Antiresorptive treatment *(drug code)*	82065998, 81256998, 81255998, 91190996, 89518998, 91190998, 91191998, 86599998, 91190997, 91191997, 93692990, 81472998, 94276990, 93827990, 92431990, 94161990, 93610990, 94245990, 61524979, 99883979, 93828990, 99867979, 95572998, 99758998, 96764998, 97398992, 95244990, 89367998, 86562998, 86561998, 87645998, 87644998, 86079998, 86076998, 91533998, 87151998, 81270998, 91027998, 93617996, 93618996, 93618997, 93617997, 90527998, 86566998, 91028998, 87137998, 87136998, 91674998, 86564998, 86567998, 87135998, 93089979, 99357998, 84212998, 84691998, 89021998, 91764998, 90551998, 91763998, 81869998, 91764997, 91763997, 89354979, 92813997, 93402998, 92813998, 98527996, 93403996, 98527998, 93403998, 93402996, 84531998, 58602979, 87606998, 85936998, 81112998, 97865998, 85935998, 81111998, 76983978, 83078978

For peer review only

Appendix 2

METHODS

Potential confounders

For smoking status, alcohol use, MRC Dyspnoea scale, and a list of prescription drugs, the most recent record within 1 year (before and after) of index date were used. A BMI record within 2 years (before and after) of index date was used.

Where possible BMI was calculated from height and weight records, for patients with a missing BMI record. The BMI was subsequently categorised (underweight: <18.5 kg/m^2, normal: 18.5-<25 kg/m^2, overweight: 25-<30 kg/m^2, obese: >30 kg/m^2).

Having received at least one prescription for inhaled corticosteroids, anti-epileptics, antidepressants, oestrogen-only Hormone Replacement Therapy (HRT) and osteoporosis medications, within 1 year (before and after) of index date were considered as risk factors.

Prediction tools – Input variables

The respective variable definitions as outlined in the algorithms for the prediction tools were used.

Smoking status – In QFracture®, three current smoking categories are provided according to the number of cigarettes smoked daily[1]. To avoid the bias of categorising patients in one of the outlying categories, "current smokers" with no documented number of cigarettes smoked were assigned to the middle category "10-19 cigarettes daily" as done in a recent publication [2]. For FRAX®'s two-category smoking status, former smokers were assigned to the "non-smoker" category as was done in the cohorts used to develop FRAX®.[3]

Alcohol consumption – similarly, for alcohol use in QFracture®, alcohol drinkers with no documented unit/day intake were assigned to "moderate (3-6units/day)".

Missing values for BMI, smoking status, and alcohol use were imputed by multiple imputation using all predictors, resulting in twenty imputed datasets.[4] A complete case sensitivity analysis without imputed variables was also performed (Appendix 3).

For peer review only

References

1 ClinRisk Ltd. QFracture-2016® risk calculator. http://www.qfracture.org/ (accessed 20 Sep 2017).

2 Dagan N, Cohen-Stavi C, Leventer-Roberts M, *et al.* External validation and comparison of three prediction tools for risk of osteoporotic fractures using data from population based electronic health records: retrospective cohort study. *BMJ* 2017;**356**:i6755. doi:10.1136/BMJ.I6755

3 Kanis JA, Oden A, Johnell O, *et al.* The use of clinical risk factors enhances the performance of BMD in the prediction of hip and osteoporotic fractures in men and women. *Osteoporos Int* 2007;**18**:1033–46. doi:10.1007/s00198-007-0343-y

4 Horton NJ, Lipsitz SR. Multiple Imputation in Practice. *Am Stat* 2001;**55**:244–54. doi:10.1198/000313001317098266

For peer review only

Appendix 3

Fracture risk prediction tools in COPD (Complete case analysis)

Of the 72,559 patients aged 40-90 years with COPD and no prior diagnosis of osteoporosis or prescription of any anti-resorptive treatment, 41,879 (57.7%) of patients had complete data. Amongst the patients with complete data, 2,649 (6.3%) experienced a MOF and 806 (1.9%) experienced hip fracture.

Both risk tools had about the same discriminatory accuracy as that obtained from the entire cohort with imputed data. The AUC for hip fracture was 75.6%, 95% CI 74.0% to 77.1% for FRAX® and 75.6%, 95% CI 74.0% to 77.2% for QFracture®. FRAX® maintained a higher accuracy for MOF (71.6%, 95% CI 70.6% to 72.6%) than QFracture® (61.1%, 95% CI 60.0% to 62.2%).

For peer review only

Appendix 4

Figure E1: Study population flow diagram

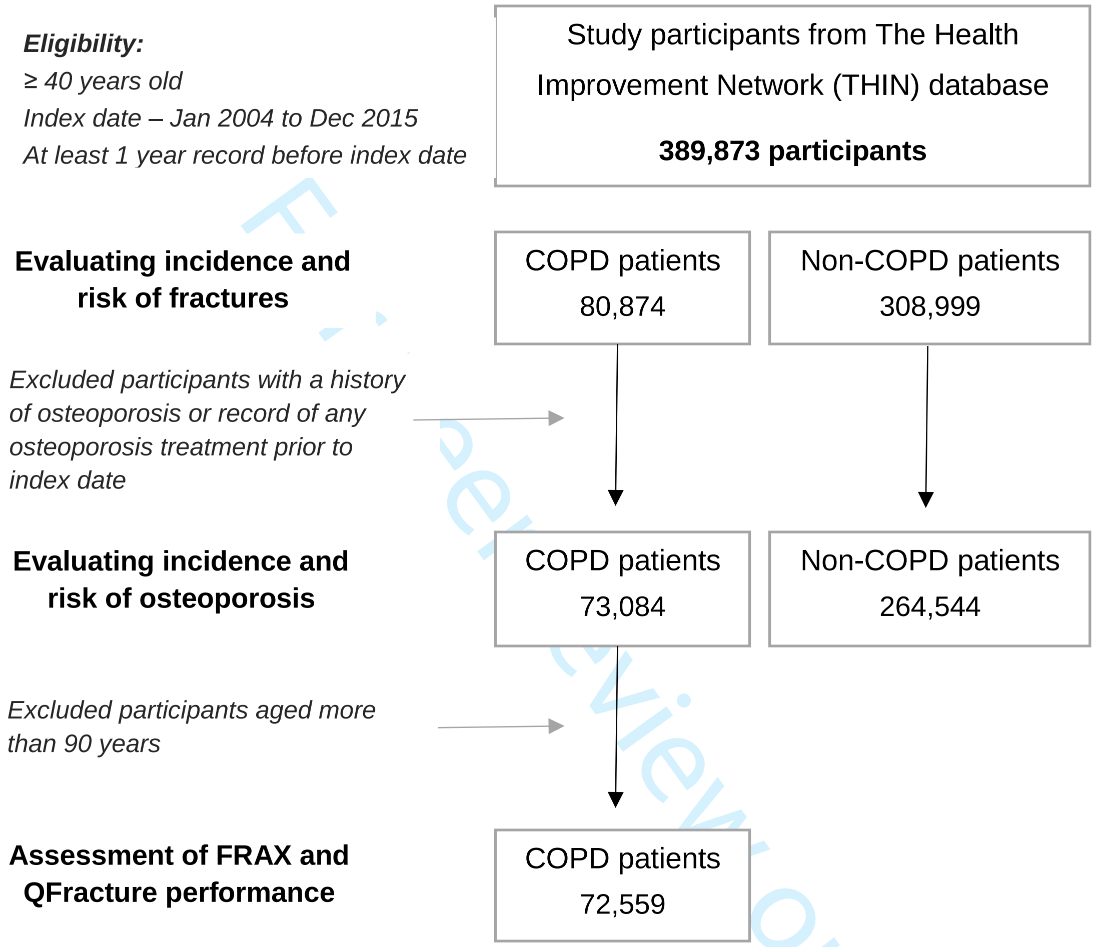

Appendix 5

Table E1: Risk of osteoporosis in patients with COPD compared with non-COPD patients

Descriptor	HR (95% CI)	Fully adjusted HR (95% CI)
**COPD**		
Non-COPD subjects	Reference	Reference
COPD patients	1.96 (1.87 – 2.06)	1.13 (1.05 – 1.22)
**Charlson Comorbidity Index**		
Score 0	Reference	Reference
Score 1	1.27 (1.18 – 1.36)	1.14 (1.06 – 1.23)
Score 2	1.34 (1.24 – 1.44)	1.27 (1.17 – 1.37)
Score 3	1.41 (1.28 – 1.55)	1.29 (1.17 – 1.42)
Score 4 & more	1.48 (1.33 – 1.64)	1.44 (1.29 – 1.61)
**Body Mass Index** (kg/m^2)		
Underweight (<18.5)	1.93 (1.64 – 2.27)	1.91 (1.63 – 2.25)
Normal (18.5 – 24.9)	Reference	Reference
Overweight (25 – 29.9)	0.64 (0.60 – 0.69)	0.63 (0.58 – 0.67)
Obese (≥ 30)	0.47 (0.43 – 0.51)	0.45 (0.41 – 0.48)
No record	0.50 (0.46 – 0.53)	0.57 (0.52 – 0.61)
**Smoking status**		
Never	Reference	Reference
Ex	1.01 (0.95 – 1.08)	1.02 (0.95 – 1.09)
Current	1.23 (1.13 – 1.33)	1.15 (1.06 – 1.25)
Unknown	0.69 (0.64 – 0.74)	0.77 (0.71 – 0.83)
**Oral Corticosteroid Use**		
Unexposed	Reference	Reference
Exposed	2.79 (2.56 – 3.05)	1.91 (1.73 – 2.10)
**Inhaled Corticosteroid Use**		
No	Reference	Reference
Yes	1.35 (1.26 – 1.45)	1.24 (1.15 – 1.34)

HR – Hazard ratio; CI – Confidence interval

HR – Cox regression model derived HR adjusted for age, sex, and GP practice

The fully adjusted Hazard Ratio (aHR) was 1.13, 95% CI 1.05 to 1.22, p<0.0001 – the multivariable Cox regression model derived aHR was adjusted for age, sex, GP practice, Charlson comorbidity index, body mass index, smoking status, inhaled corticosteroid use, and cumulative oral corticosteroid use.

For peer review only

Appendix 6

Table E2: Baseline characteristics of patients with COPD aged 40-90 years with no prior diagnosis of osteoporosis or prescription of any anti-resorptive treatment

Descriptor	COPD patients	
	n = 72,559	%
**Mean age at index date (years, SD)**	66.1 (10.7)	
**Sex**		
Female	31,885	43.9
**MRC Dyspnoea Scale** (1 Year either side of diagnosis)		
1	8,882	12.2
2	17,718	24.4
3	9,257	12.8
4 & 5	4,346	6.0
No record	32,356	44.6
**Charlson Comorbidity Index Score**		
0	0	0
1	38,573	53.2
2	11,953	16.5
3	11,110	15.3
≥ 4	10,923	15.1
**Body Mass Index (BMI) (kg/m²)**		
Underweight (< 18.5)	2,730	3.8
Normal (18.5 – 24.9)	21,791	30.0
Overweight (25 – 29.9)	21,504	29.6
Obese (≥30)	17,627	24.3
No BMI	8,907	12.3
**Smoking status** (1 Year either side of diagnosis)		
Never smoked	7,062	9.7
Ex-smoker	33,810	46.6
Current smoker	29,949	41.3
Unknown	1,738	2.4

For peer review only

STROBE 2007 (v4) Statement—Checklist of items that should be included in reports of *cohort studies*

Section/Topic	Item #	Recommendation	Reported on page #
**Title and abstract**	1	(*a*) Indicate the study's design with a commonly used term in the title or the abstract	1
		(*b*) Provide in the abstract an informative and balanced summary of what was done and what was found	2
**Introduction**			
Background/rationale	2	Explain the scientific background and rationale for the investigation being reported	5
Objectives	3	State specific objectives, including any pre-specified hypotheses	5
**Methods**			
Study design	4	Present key elements of study design early in the paper	6
Setting	5	Describe the setting, locations, and relevant dates, including periods of recruitment, exposure, follow-up, and data collection	6
Participants	6	(*a*) Give the eligibility criteria, and the sources and methods of selection of participants. Describe methods of follow-up	6
		(*b*) For matched studies, give matching criteria and number of exposed and unexposed	6
Variables	7	Clearly define all outcomes, exposures, predictors, potential confounders, and effect modifiers. Give diagnostic criteria, if applicable	6,7
Data sources/ measurement	8*	For each variable of interest, give sources of data and details of methods of assessment (measurement). Describe comparability of assessment methods if there is more than one group	6,7
Bias	9	Describe any efforts to address potential sources of bias	7
Study size	10	Explain how the study size was arrived at	-
Quantitative variables	11	Explain how quantitative variables were handled in the analyses. If applicable, describe which groupings were chosen and why	7
Statistical methods	12	(*a*) Describe all statistical methods, including those used to control for confounding	7
		(*b*) Describe any methods used to examine subgroups and interactions	-
		(*c*) Explain how missing data were addressed	6, Appendix 2
		(*d*) If applicable, explain how loss to follow-up was addressed	-
		(*e*) Describe any sensitivity analyses	7
**Results**			
Participants	13*	(a) Report numbers of individuals at each stage of study—eg numbers potentially eligible, examined for eligibility, confirmed	9

For peer review only

		eligible, included in the study, completing follow-up, and analysed	
		(b) Give reasons for non-participation at each stage	-
		(c) Consider use of a flow diagram	-
Descriptive data	14*	(a) Give characteristics of study participants (eg demographic, clinical, social) and information on exposures and potential confounders	9, Table 1 (17)
		(b) Indicate number of participants with missing data for each variable of interest	Table 1 (19)
		(c) Summarise follow-up time (eg, average and total amount)	9
Outcome data	15*	Report numbers of outcome events or summary measures over time	9, 10
Main results	16	(*a*) Give unadjusted estimates and, if applicable, confounder-adjusted estimates and their precision (eg, 95% confidence interval). Make clear which confounders were adjusted for and why they were included	9, 10, Table 2 (20), Appendix 5
		(*b*) Report category boundaries when continuous variables were categorized	Appendix 2
		(*c*) If relevant, consider translating estimates of relative risk into absolute risk for a meaningful time period	-
Other analyses	17	Report other analyses done—eg analyses of subgroups and interactions, and sensitivity analyses	9,10,11
**Discussion**			
Key results	18	Summarise key results with reference to study objectives	12
**Limitations**			
Interpretation	20	Give a cautious overall interpretation of results considering objectives, limitations, multiplicity of analyses, results from similar studies, and other relevant evidence	12-14
Generalisability	21	Discuss the generalisability (external validity) of the study results	14
**Other information**			
Funding	22	Give the source of funding and the role of the funders for the present study and, if applicable, for the original study on which the present article is based	15

*Give information separately for cases and controls in case-control studies and, if applicable, for exposed and unexposed groups in cohort and cross-sectional studies.

Note: An Explanation and Elaboration article discusses each checklist item and gives methodological background and published examples of transparent reporting. The STROBE checklist is best used in conjunction with this article (freely available on the Web sites of PLoS Medicine at http://www.plosmedicine.org/, Annals of Internal Medicine at http://www.annals.org/, and Epidemiology at http://www.epidem.com/). Information on the STROBE Initiative is available at www.strobe-statement.org.

For peer review only
